# App-Based Physical Activity Intervention for Individuals With Depression (MoodMover): Single-Arm, Pre-Post Proof-of-Concept and Feasibility Study

**DOI:** 10.2196/79033

**Published:** 2026-06-11

**Authors:** Yiling Tang, Madelaine Gierc, Sam Liu, Raymond W Lam, Eli Puterman, Guy Faulkner

**Affiliations:** 1 School of Kinesiology, The University of British Columbia, 6081 University Blvd, Vancouver, BC, V6T 1Z1, Canada, 1 2369677785; 2 Department of Sports and Health Sciences, Academy of Wellness and Human Development, Hong Kong Baptist University, Hong Kong, China; 3 School of Exercise Science, Physical & Health Education, University of Victoria, Victoria, BC, Canada; 4 Department of Psychiatry, The University of British Columbia, Vancouver, BC, Canada

**Keywords:** mobile apps, physical activity, depression, mHealth, behavior change, feasibility

## Abstract

**Background:**

Depression is a prevalent mental disorder, and it remains one of the leading causes of disability in Canada and globally. Mobile app–based physical activity interventions may offer an effective and accessible treatment option for individuals with depression who cannot or prefer not to access supervised exercise programs.

**Objective:**

This study aims to investigate the feasibility, acceptability, and proof of concept of a 9-week, theory-guided, app-based physical activity promotion intervention (MoodMover) developed for people with depression.

**Methods:**

We conducted a single-arm, pre-post study from November 2024 to May 2025, following the phase IIa: Proof-of-concept and phase IIb: Pilot and Preliminary Testing of the Obesity-Related Behavioural Intervention Trials model. Physically inactive adults who either self-reported a diagnosis of major depressive disorder or reported at least mild depressive symptoms, operationalized as a minimum score of 5 on the Patient Health Questionnaire, 9-Item, were recruited. The intervention spanned 9 weeks, with the first week serving as a run-in period and including a 15-minute orientation session on the first day. Participants were instructed to use the MoodMover program, delivered via the Pathverse app. Feasibility was assessed based on 4 primary criteria: recruitment, adherence, usability, and retention. Proof-of-concept was evaluated by assessing changes in physical activity behavior and depressive symptoms over the intervention period.

**Results:**

From November 2024 to March 2025, 32 of the 51 adults who met eligibility criteria consented to participate in this study, resulting in a recruitment rate of 63%. Twenty-eight participants completed baseline assessments, with a mean age of 39.8 (SD 13.4) years. A total of 21 participants attended the orientation session and received the intervention. Retention, adherence, and usability rates were 57% (16/28), 67% (14/21), and 50% (8/16), respectively. Regression analyses found that age consistently associated with app engagement, usability, and satisfaction. Two-tailed paired *t* tests indicated significant pre-post changes in self-reported moderate to vigorous physical activity and depressive symptoms across the 75%, 85%, and 95% CIs. Among participants with clinically elevated depressive symptoms at baseline (Patient Health Questionnaire, 9-Item ≥10), 75% (9/12) achieved a clinically meaningful reduction in symptom severity.

**Conclusions:**

Our findings suggest that MoodMover holds potential for promoting physical activity behavior among individuals with depression and supporting depression management at scale. However, the feasibility of the tested version remains suboptimal. Necessary modifications (eg, improvements to enhance the accuracy of step tracking) should be implemented and reevaluated before progressing to a more rigorous efficacy trial.

## Introduction

Depression is the largest contributor to disability, affecting approximately 280 million people worldwide [1]. It has been linked with a higher incidence of various noncommunicable diseases such as cardiovascular diseases [2], type II diabetes [3], pulmonary disorders [4], and 10‐14 years of premature mortality [5]. Approximately 60% of those with major depressive disorder (MDD) are diagnosed with comorbidities, contributing to an increased disease burden worldwide [6]. In Canada, the burden is substantial: according to the most recent 2022 Mental Health and Access to Care Survey, the 12-month prevalence of MDD was 7.6%, and the lifetime prevalence was 14.0% [7]. The annual economic burden of depression in Canada is estimated to be more than CAD $12 billion (approximately US $8.6 billion) for the health care system [8], a figure that accounts for direct medical costs (eg, hospitalizations, physician visits, and prescription drugs), as well as social service costs (eg, employment assistance).

Despite the increased availability of many evidence-based treatments (eg, psychotherapy and antidepressants) over recent decades [9], access to these first-line treatments remains low globally due to various barriers. These include concerns about the side effects of antidepressants, limited resources, a critical shortage of health care professionals, and the stigma associated with seeking mental health care [1]. In Canada, more than half of the people seeking mental health care reported unmet needs, particularly for counseling or therapy [10]. More discouragingly, when Ormel et al [9] sought to explain the "more treatment but no less depression paradox," they proposed three primary explanations: (1) the published literature tends to overestimate the short- and long-term efficacy of treatments, (2) the effectiveness of treatments is notably reduced when applied in real-world settings, and (3) the impact of treatment varies significantly between chronic recurrent and nonrecurrent cases. Taken together, these findings underscore the urgent need for effective complementary mental health interventions that are both more accessible and effective in real-world contexts [11].

Physical activity (PA) is widely recognized as a modifiable health behavior that can help prevent and manage depression [12-14]. It has also been consistently associated with various other physical and mental health benefits, such as improved sleep quality, prevention of multiple cancers, and reduced anxiety [15,16]. Based on the existing evidence, exercise has been recommended as a treatment for depression in Canada by both the 2016 and 2023 Canadian Network for Mood and Anxiety Treatments (CANMAT) guidelines [17,18] and in the United Kingdom by the National Institute for Health and Care Excellence guidelines [19]. However, exercise remains an underutilized depression treatment option [20]. There is now a critical need to translate guidelines into practice by developing accessible, evidence-based options to support individuals with depression in increasing their PA levels.

To enhance the accessibility of such interventions, mobile apps have emerged as a widely adopted mode of delivery [21,22]. MoodMover, a 9-week smartphone app–based intervention, was developed to promote PA behavior change and potentially alleviate depressive symptoms. The intervention is theoretically grounded in the Multi-Process Action Control (M-PAC) framework [23] and integrates two systematic behavioral intervention development frameworks to achieve synergistic effects: (1) the Obesity-Related Behavioural Intervention Trials (ORBIT) [24] for an ultimate goal of managing chronic disease, and (2) the Integrate, Design, Assess, and Share [25] model for the enhancement of digital intervention development.

The detailed, step-by-step development process and usability testing of the MoodMover prototype are described elsewhere [26]. This study aligns with phase IIa: Proof-of-concept and phase IIb: Pilot and Feasibility Testing of the ORBIT model. This study aimed to determine the feasibility and acceptability of the current version of MoodMover in people with depression, along with its preliminary effects on behavioral (ie, daily step counts) and clinical outcomes (ie, depressive symptoms). The findings will inform whether the intervention is ready for evaluation in a more rigorous study, that is, ORBIT phase IIc.

## Methods

### Study Design

This study was a single-arm pre-post trial. The full protocol for this trial has been previously published and contains a detailed description of the methods used, study outcomes, and progression criteria [27]. This trial is registered at ClinicalTrials.gov (NCT06573125). The reporting of this study followed a guide proposed by Lancaster and Thabane [28], recommending the use of the STROBE (Strengthening the Reporting of Observational Studies in Epidemiology) statement alongside the CONSORT (Consolidated Standards of Reporting Trials) extension for pilot and feasibility trials (Checklist 1).

### Ethical Considerations

Ethics approval was obtained from The University of British Columbia Behavioural Research Ethics Board (H24-01820). In accordance with ethics approval, participants were given 1 week to review the study materials, after which a follow-up email was sent to confirm their interest. Eligible volunteers who agreed to participate completed the informed consent process electronically; only electronic signatures were obtained, as approved by the research ethics board. Participants could withdraw from the treatment or the study at any time. All data were collected and stored on secure, password-protected platforms, with access limited to authorized research team members. To protect participant privacy, no identifying information (eg, names, initials, or contact details) was disclosed in any publication or presentation arising from this study. All data reported are anonymized, and no images or other materials that could identify individual participants are included in this manuscript or supplementary files. Participants were compensated with a CAD $10 (approximately US $7.00) Amazon e-gift card for completing assessments at each time point.

### Intervention

The MoodMover intervention is a 9-week, self-guided program accessible via the Pathverse app [29], available on both iPhone (Apple Inc) and Android devices. The program begins with a 1-week run-in period to collect baseline step data, followed by an 8-week intervention phase, and concludes with an optional 9-week follow-up period. During the intervention phase, participants receive 2 psychoeducational lessons per week: 1 major and 1 optional complementary lesson, released at prescheduled times. Various formats are used to deliver the educational content, including podcasts, GIFs, and illustrated texts. In addition, MoodMover incorporates a gamification component based on lesson completion, awarding 20 and 10 points for completing major and complementary lessons, respectively. For every 60 points accumulated, participants receive a CAD $5 (approximately US $3.50) Amazon e-gift card.

The program uses a progressive step goal-setting strategy, encouraging participants to increase their daily step count by 1000 steps above their baseline (established during the run-in week), with additional 1000-step increments every 2 weeks, ultimately targeting a total increase of 3000 steps per day [26,30,31]. The program is embedded with diverse features, operationalizing a range of behavior change techniques (BCTs) [32] to apply the theoretical constructs defined by the M-PAC model. A list of unique BCTs used in this version of MoodMover is provided in Multimedia Appendix 1. Coding was conducted independently by 2 coders (YT and VW) using the BCT Taxonomy version 1 (BCTTv1) [32], with discrepancies resolved through discussion.

### Participants

Participants were required to be adults aged 18‐64 years with at least mild depressive symptoms, defined as meeting either of the following: (1) self-reported a current diagnosis of MDD, or (2) scored ≥5 on the Patient Health Questionnaire, 9-Item (PHQ-9) [33]. In practice, all participants who self-reported a diagnosis also scored ≥5 on the PHQ-9. All participants were smartphone owners (either iPhone or Android), demonstrated literacy in English, had an active email address, and were physically inactive (self-reported less than 90 minutes of moderate to vigorous PA [MVPA] per week). Concomitant care and interventions were permitted, provided that their dosage remained stable throughout the program. Individuals who met any of the following criteria were excluded: self-reported physical disability or health condition that prevents exercise, self-reported active psychosis or mania, active suicidal thoughts (PHQ-9 score ≥1), severe cognitive impairment, being currently pregnant, or expected a major absence during the intervention. All participants provided informed consent before initiating the study [34].

### Recruitment

Participants were recruited from November 15, 2024, to March 26, 2025. Recruitment was conducted remotely to reflect real-world conditions and used a multipronged, systematic approach. First, an electronic study posting was placed on REACH BC [35], a provincial research study recruitment database. Second, recruitment posters were displayed in 3 community spaces, with permission from site administrators. Third, we contacted a total of 28 local nonprofit mental health associations and free public depression advocacy groups to request dissemination of study information. Outreach was conducted via web form, email, telephone, and in-person visits using publicly available contact information, as well as through professional networking. To mimic real-world conditions, recruitment strategies were implemented in a staggered manner rather than simultaneously, and they were adjusted over time based on available resources and the pace of enrollment. Specifically, the electronic posting was initiated first; outreach to mental health associations and advocacy groups began 1 week later and continued throughout the recruitment period, while paper posters were displayed at 2 weeks and 1 month after the start of recruitment.

Interested volunteers were directed to an eligibility screening questionnaire on REDCap (Research Electronic Data Capture; see Multimedia Appendix 2 for all questionnaires used in this study). Following completion of the screening, individuals who were ineligible due to active suicidal ideation were immediately notified via email and provided with community mental health resources. Eligible individuals were invited to participate and received detailed study information electronically.

### Procedures

After providing informed consent, the researcher (YT) registered participants who completed the preintervention assessments on Pathverse. Participants were then provided with a MoodMover user guide and an instructional letter outlining preparatory tasks for the orientation session. On the first day, participants attended a 15-minute one-on-one orientation via Zoom (Zoom Communications, Inc) on their smartphones. Five orientation sessions were recorded. According to the study protocol, orientation session recordings were to be transcribed immediately and assessed by a research assistant using a fidelity checklist to enable timely modifications for improving treatment fidelity after each session [36]. However, this protocol was not feasible due to the short intervals (eg, less than 1 hour) between sessions. As an alternative, the researcher (YT) took notes after each orientation session, cross-checked them with the fidelity checklist, and implemented improvements in subsequent sessions. The recordings were later reviewed to verify the accuracy and completeness of the fidelity assessments based on the session notes.

After completing the intervention, participants were invited to complete postintervention assessments on REDCap. Along with the invitation, participants were reminded of the optional follow-up, which included continued tracking of their app engagement and step data for an additional 9 weeks. Participants who chose not to continue could either delete the app or inform the researcher (YT) of their decision to withdraw. Those who opted in were approached on the final day of the follow-up period to complete an intervention satisfaction questionnaire and sync their step data with MoodMover, allowing for PA data collection throughout the entire follow-up period. All procedures were conducted entirely remotely.

### Measures

A set of measures was used to assess primary feasibility and acceptability outcomes, secondary behavioral and clinical outcomes, and key demographic information. Further details on all assessment tools are available in the published protocol paper [27].

#### Demographic and Clinical Information

Demographic data and clinical information, such as sex and gender, race or ethnicity, age, other ongoing treatments relevant to depression management, substance use (eg, cannabis), and current and past usage of PA apps or devices, were collected.

#### Primary Feasibility and Acceptability Outcomes

The traffic light system [37] was used to determine progression to a randomized controlled trial (RCT) for efficacy testing. The decision was based on the lowest performing criterion among the following metrics: (1) recruitment, (2) adherence, (3) usability, and (4) retention (Table 1).

**Table 1.** Predetermined feasibility progression criteria for MoodMover for adults with depression.

	Definition	Green zone	Yellow zone	Red zone
Action		Proceed with no changes	Proceed with amendments	Stop intervention development
Metric				
Recruitment	Proportion of eligible participants who provided consent	≥65%	40%‐64%	≤39%
Adherence	Proportion of participants who completed ≥5 major lessons	≥70%	40%‐69%	≤39%
Usability	Proportion of participants reporting a score of ≥5 on the MAUQ^a^	≥70%	40%‐69%	≤39%
Retention	Proportion of participants who completed the baseline questionnaire	≥70%	40%‐69%	≤39%

a MAUQ: mHealth app usability questionnaire.

#### Recruitment

The recruitment rate was defined as the proportion of eligible individuals who provided consent in the program. Additionally, the recruitment duration, sources, and the number of sites where posters were displayed were documented.

#### Adherence

Intervention adherence was defined as the proportion of participants completing at least 5 of the 8 major lessons. A few additional engagement metrics, such as average time spent on major and complementary modules and total app use duration (excluding time spent on features under the menu tab), were extracted from the Pathverse admin portal. Participants were classified as “complete users” (all 8 major modules completed), “incomplete users” (2‐7 modules completed), or “nonusers” (no app use after orientation session). Participants inactive for 2 weeks were contacted via email for a check-in, and any reported reasons for nonadherence were documented.

#### Usability

The adapted patient version of the mHealth app usability questionnaire (MAUQ) [26,38] for stand-alone apps was used, consisting of 13 items. Participants rated each item on a 7-point Likert scale (1=strongly disagree, 7=strongly agree). An average score of 5 or higher was considered indicative of acceptable usability [26].

#### Retention

Retention was defined as the proportion of participants who completed both pre- and postintervention assessments. Reasons for early discontinuation (eg, lack of time, technical issues, and personal reasons) and any reported adverse events were documented.

#### Compliance, Satisfaction, and Acceptability of Outcome Measures

Compliance with the graded goal-setting protocol and user satisfaction were evaluated as indicators of feasibility and acceptability. Participants who followed the goal-setting instructions at all designated time points (weeks 2, 4, and 6) were classified as having “full compliance.” Those who followed instructions at 1 or 2 time points were considered to have “partial compliance,” while those who did not follow instructions at any time were categorized as having “no compliance.” User satisfaction was measured using the mHealth Satisfaction Questionnaire [39], with scores ranging from 14 to 70, where higher scores indicate greater satisfaction. Participants were also asked to list 3 things they liked and 3 things they disliked about the intervention. The response rate and the amount of complete data for self-reported outcomes were reported.

### Secondary Outcomes

#### PA (Behavioral Outcome)

Daily step counts were obtained from the smartphone’s built-in step-tracking feature and downloaded via the Pathverse admin web portal. Self-reported PA was assessed using the Canadian Physical Activity Adult Questionnaire [40] at both baseline and postintervention. In addition, participants indicated whether they had connected a smartwatch or fitness tracker to Health (iOS) or Google Fit (Android) throughout the program. They also reported the frequency (almost always, sometimes, or seldom) with which they carried their phone or wore their smartwatch during nonsedentary waking hours on both weekdays and weekends.

#### Depressive Symptoms (Clinical Outcome)

The PHQ-9 was used to measure self-reported depressive symptoms [33]. Participants rated 9 items on a 0-3 Likert scale, yielding a total score between 0 and 27, with higher scores indicating greater symptom severity. Depression severity was classified as follows: 5‐9 (mild), 10‐14 (moderate), 15‐19 (moderately severe), and 20‐27 (severe) [41]. A clinically meaningful improvement was defined by McMillan et al [42] as a 5-point reduction on the PHQ-9 and a shift from a baseline score of ≥10 to ≤9 post intervention. As individuals with mild depressive symptoms were eligible for this study, this criterion was applied only to those participants with baseline scores of 10 or higher.

#### Anxiety

Anxiety symptoms were assessed using the Generalized Anxiety Disorder, 7-Item [43]. The Generalized Anxiety Disorder, 7-Item consists of 7 items rated on a 0-3 Likert scale [44], with a maximum score of 21. Higher scores indicate more severe anxiety. Anxiety severity was classified as follows: 5‐9 (mild), 10‐14 (moderate), and 15‐21 (severe) [44].

#### Sleep Quality

The 19-item Pittsburgh Sleep Quality Index [45] was used to assess sleep quality. Participants rated items on a 0-3 Likert scale, with scores summed across 7 components (eg, sleep duration and sleep latency) to yield a total score ranging from 0 to 21. Higher scores indicate poorer sleep quality, with a total score greater than 5 signifying poor sleep quality.

#### Functional Disability

World Health Organization Disability Assessment Schedule 2.0 [46] was used to evaluate participants’ health and ability to perform activities across 6 functional areas (eg, cognition and mobility). The World Health Organization Disability Assessment Schedule 2.0 consists of 12 items, where participants estimate the extent of their disability over the past 30 days using a 5-point Likert scale. The total score ranges from 0 to 100, with higher scores indicating greater disability.

#### M-PAC Constructs

Items to assess M-PAC constructs were primarily derived from Tang et al [47], Rhodes and Lim [48], and Wilson and Muon [49]. *Affective* and *instrumental attitudes* were assessed using three 7-point bipolar scales. *Perceived opportunity* and *capability* were evaluated with three 5-point Likert scales, ranging from 1 (strongly disagree) to 5 (strongly agree). *Intention strength* was measured with 3 items (eg, “I am committed to engaging in physical activity over the next month”), rated on a 5-point Likert scale from 1 (strongly disagree) to 5 (strongly agree). Additionally, *decisional intention* was assessed with 1 item regarding the goal to increase daily steps by 3000 above baseline for most days per week. Participants responded with a binary (yes or no) answer. *Regulations* were measured using 5-point Likert scales, with 3 items related to self-monitoring, goal-setting, and action planning. Both *habit* and *identity* were measured with 3 items on a 5-point Likert scale, respectively. Further details are described in the protocol [27].

### Sample Size Justification

Overall, a pragmatic sample size range of 20‐36 participants was estimated and adopted, following commonly accepted sample size rule of thumb for feasibility trials [50,51] and recommendations for pilot RCT intervention arms using the “traffic light” progression criteria [52].

### Statistical Analysis

All analyses were performed using SPSS (version 29; IBM Corp) or R (version 4.2.2; R Core Team) via RStudio (version 2024.12.0+467; Posit PBC). Descriptive statistics were presented for all measures. The strategies for addressing missing data were determined by evaluating the degree and pattern of missingness. When reporting the results, participants were grouped into (1) study completers, defined as those who remained enrolled through week 9, regardless of their level of weekly engagement; and (2) noncompleters, which included nonstarters (those who enrolled but did not attend the orientation session) and early dropouts (those who used the app briefly before discontinuing). Regression-based analyses were used to explore the influence of potential participant-level factors (eg, sex and age) on feasibility results, with separate models for each factor. Unstandardized regression coefficients (*B*) are reported for all regression analyses. Changes in secondary outcomes (eg, PA, depression, and M-PAC constructs) over time were assessed using 2-tailed paired *t* tests for continuous data, Wilcoxon signed-rank tests for nonnormal distributions, and McNemar test for binary outcomes. Corrections for multiple comparisons were not applied, as the analyses were exploratory in nature. Pearson and Spearman correlation analyses were conducted to examine the associations between changes in PA and changes in depressive symptoms. The results of secondary outcomes were reported with 75%, 85%, and 95% CIs for estimation purposes [53,54]. The predetermined clinically meaningful decrease in depression was also incorporated into the interpretation for those with baseline PHQ-9 scores of 10 or higher.

## Results

### Recruitment

**Figure 1.**
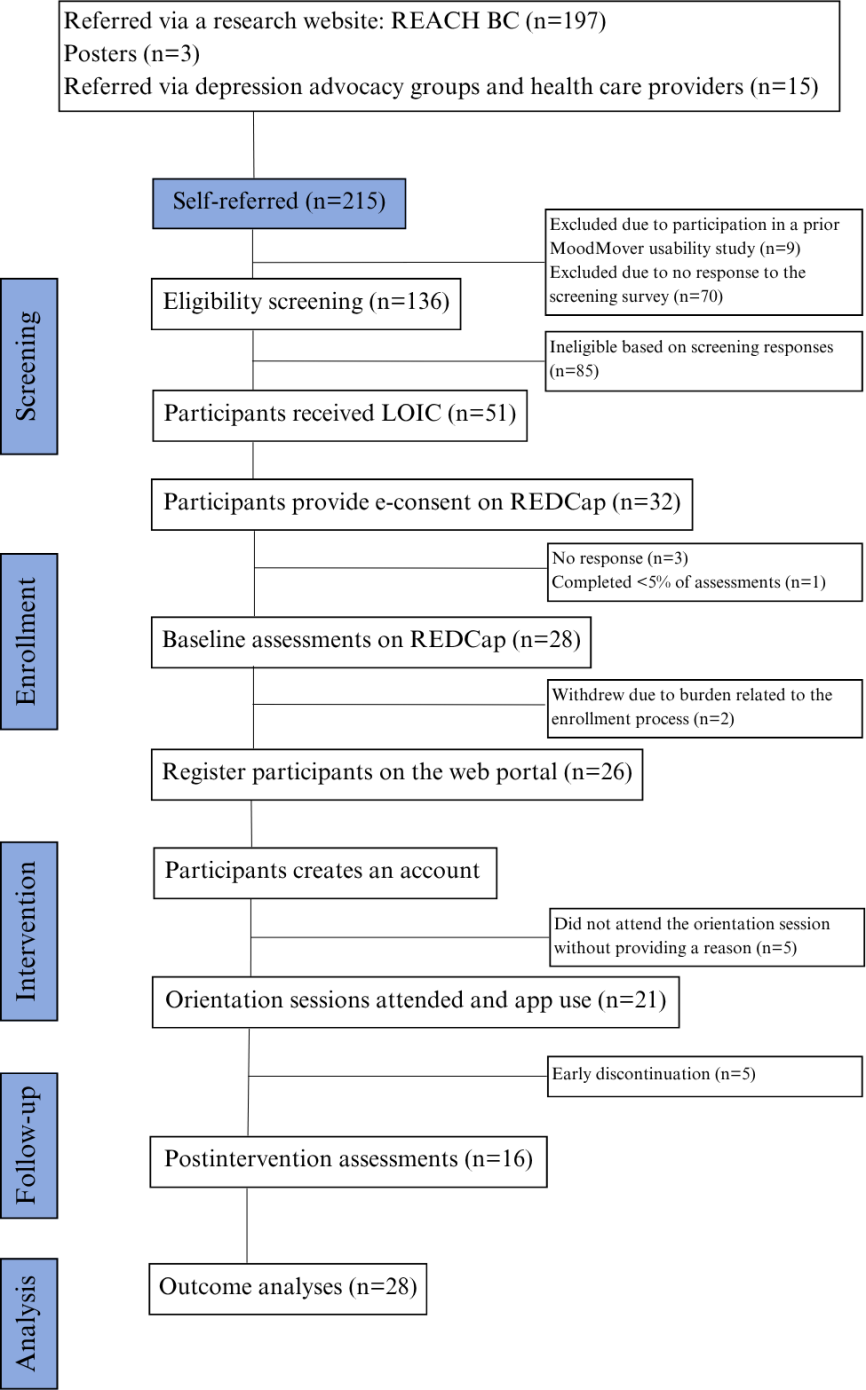
Participant flow diagram for a proof-of-concept and feasibility study of MoodMover for adults with depression. LOIC: Letter of Information and Consent; REDCap: Research Electronic Data Capture.

Of the 215 adults who self-referred to MoodMover (Figure 1), 197 (91.6%) were recruited through REACH BC. During the first month, recruitment progressed at a promising pace: 122 individuals expressed interest, 75 completed the screening survey, 27 were deemed eligible, and 16 enrolled. However, outreach to advocacy groups was less effective, with 0 of the 15 contacted groups responding. Recruitment slowed during the holiday period (December 15, 2024, to January 5, 2025) and declined further after 2 months. In response, additional advocacy groups were contacted in January and February, with 4 out of 13 responding, resulting in 15 referrals. An additional 3 participants were recruited through posters.

Nine potential participants were deemed ineligible due to their prior participation in the previous usability study [26]. A total of 70 participants did not finish the screening process. Of the remaining 136 individuals screened, 51 physically inactive adults with at least mild depression symptoms were deemed eligible. A total of 32 participants provided informed consent, resulting in a recruitment rate of 63% (32/51), which was slightly below the predefined progression criterion threshold (>65%).

### Baseline Characteristics

Three participants did not initiate the preintervention assessments, and 1 participant initiated but completed less than 5% of items. This resulted in a preintervention response rate of 91% (29/32) and a preintervention completion rate of 88% (28/32).

Multimedia Appendix 3 presents baseline characteristics of the 28 participants and compares study completers with noncompleters. The participants had a mean age of 39.8 (SD 13.4) years. Completers appeared older than noncompleters (44.1 vs 34.0 years), although no statistical comparisons were conducted. Out of 28 participants, 20 (71%) self-identified as women, 4 (14%) as men, 3 (11%) as nonbinary, and 1 (4%) preferred not to answer. Most participants were White (18/28, 64%), and half had obtained a bachelor’s degree or higher. Out of 28 participants, 17 (61%) self-reported a diagnosis of MDD, with most of them (13/17, 77%) having had the diagnosis for more than 2 years. Most participants (24/28, 86%) were currently taking antidepressants, with the majority of those (14/24, 58%) having been on them for more than 2 years. Out of 28 participants, 23 (82%) were currently using or had previously used other PA apps or devices.

### Feasibility and Acceptability

Feasibility and acceptability results are presented in Multimedia Appendix 4.

#### Adherence

A total of 21 participants attended orientation sessions and received the intervention. Intervention adherence, defined as the proportion of participants completing at least 5 major lessons, was 67% (14/21), falling within the amber zone of the predetermined progression criterion. No participants were classified as “nonusers” (ie, no app use following the orientation session). In total, 48% (10/21) of the participants were identified as “complete users,” having completed all major lessons, while the remaining 52% (11/21) of the participants were classified as “incomplete users,” with a mean of 3.6 (SD 2.1) major lessons completed. Among those who met the adherence criterion, the average number of completed major lessons was 7.4 (SD 1.2).

**Figure 2.**
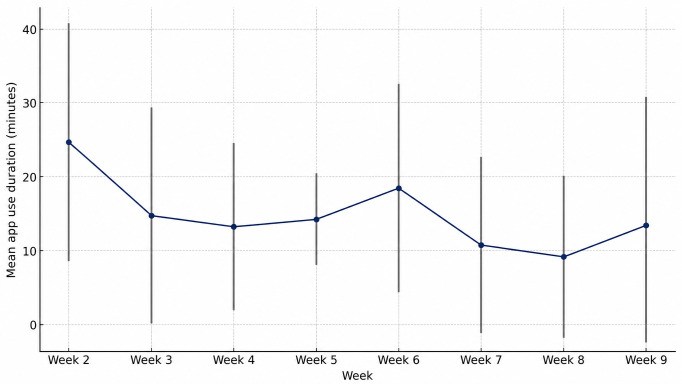
Weekly app use duration during the intervention phase among participants in a proof-of-concept and feasibility study of MoodMover for adults with depression. Time spent on features under the menu tab, including Q&A, resources, survey history, and my points, is not included due to data unavailability.

On average, participants spent 8.0 (SD 4.7) minutes and 3.9 (SD 3.0) minutes on each major and complementary lessons, respectively. All participants, except 1, completed the first 2 major lessons and the first complementary lesson. Throughout the 8-week intervention phase (weeks 2‐9), participants engaged with the app for an average of 119.6 (SD 66.9) minutes, with app activity recorded on 55% (30.8/56; SD 15.2) of days with at least 1 use. Figure 2 demonstrates how the duration of weekly app use changed over the intervention period (see Multimedia Appendix 5 for details). Participants synced their steps a mean of 28.5 (SD 17.1; range 0‐63) times. Most participants (19/21, 91%) synced their steps at least 12 times, except for 2 Android users who experienced serious technical issues related to the step syncing feature.

Of the 21 app users, 18 (86%) used the goal-setting feature, 11 (52%) used the action planning feature, and 17 (81%) used the exercise logging and mood tracking feature. In total, 48% (10/21) of the participants used the community forum to report technical issues (n=6) or share their exercise experiences (n=5). Participants completed a mean of 6.5 (SD 17.8; range 0‐82) action plans and self-logged an average of 6.7 (SD 9.9; range 0‐36) exercise sessions. Each participant recorded an average of 4.5 (SD 7.6) light PA sessions and 2.2 (SD 3.8) MVPA sessions, accumulating 135.7 (SD 176.7) minutes of light PA and 109.1 (SD 207.5) minutes of MVPA, respectively. Participants reported a mean mood rating of 2.7 (SD 0.4) on a 0-4 scale after exercising.

#### Compliance

Participants modified their step goals an average of 2.3 (SD 1.5; range 0‐5) times. Out of 21 participants, 9 (43%) demonstrated full compliance, adhering to all instructions across the designated time points to set new step goals. Another 9 (43%) participants were classified as partially compliant, adjusting their step goals at 1 or 2 time points. Specifically, 17 (81%) participants set a step goal in week 2, while 15 (71%) and 9 (43%) modified their goals as instructed in weeks 4 and 6, respectively. A total of 3 (14%) participants did not modify their step goals at any time and were categorized as noncompliant.

#### Retention Rates and Adverse Events

A total of 16 participants completed both pre- and postintervention questionnaires, yielding a retention rate of 57% (16/28), which falls within the amber zone of the progression criterion for retention. Reported reasons for discontinuation included being too busy (n=2), weather-related challenges (n=1), technical issues (n=1), mental health issues (n=1), and falling behind in the lessons (n=1).

One participant reported an adverse event of feeling pressured to engage in activities that did not feel natural (eg, self-set action plan reminders were intrusive due to a frequently changing schedule). No other adverse events were reported.

#### Usability and Satisfaction

Half (8/16, 50%) of the participants rated MoodMover as 5 or greater on MAUQ, with an average mean score of 5.01 (SD 1.02)—equivalent to the amber zone based on the predetermined criterion on usability. Participants rated MoodMover as having higher usability in terms of interface design and overall satisfaction (mean 5.00, SD 1.07) and usefulness (mean 5.44, SD 1.31), while lower usability scores were reported for ease of use (mean 4.94, SD 1.03). Out of 21 participants, 10 (48%) experienced varying levels of technical issues. A list of the issues encountered is presented in Multimedia Appendix 6. Users’ satisfaction levels ranged from 36 to 66, with a mean of 53.56 (SD 8.11).

### Factors Affecting App Engagement, Usability, and Satisfaction

Age was consistently associated with most app engagement metrics (ie, total app use duration and active app use days during intervention phase, major lesson completion, and average minutes spent on each major lesson), usability (MAUQ), and satisfaction metrics across the 75%, 85%, and 95% CIs. For example, the association between age and usability was *B*=0.04 (75% CI 0.02‐0.06, 85% CI 0.02‐0.07, and 95% CI 0.01‐0.08, respectively). The association between age and satisfaction was *B*=0.38 (75% CI 0.22‐0.53, 85% CI 0.18‐0.58, and 95% CI 0.10‐0.66, respectively). Additionally, participants’ BMI, baseline depression levels, and baseline self-reported MVPA levels were associated with certain app engagement, usability, and satisfaction metrics. For instance, the association between baseline depression and usability was *B*=−0.70 (75% CI −0.96 to −0.43, 85% CI −1.03 to −0.36, and 95% CI −1.17 to −0.23, respectively). Full results are presented in Multimedia Appendix 7. Results involving categorical variables should be interpreted with caution due to instability and wide variability caused by levels with very small sample sizes (n<5).

A total of 16 participants provided open-ended feedback on the features they liked and disliked about the app. The most frequently cited positive aspects included reminders (n=7), ramped goals (n=6), and lessons (n=6). Conversely, the most commonly mentioned areas for improvement were step syncing (n=9; including the need to carry their phone, n=3), navigation (n=3), exercise logging (n=3), and the user interface (n=3).

### Secondary Outcomes

**Figure 3.**
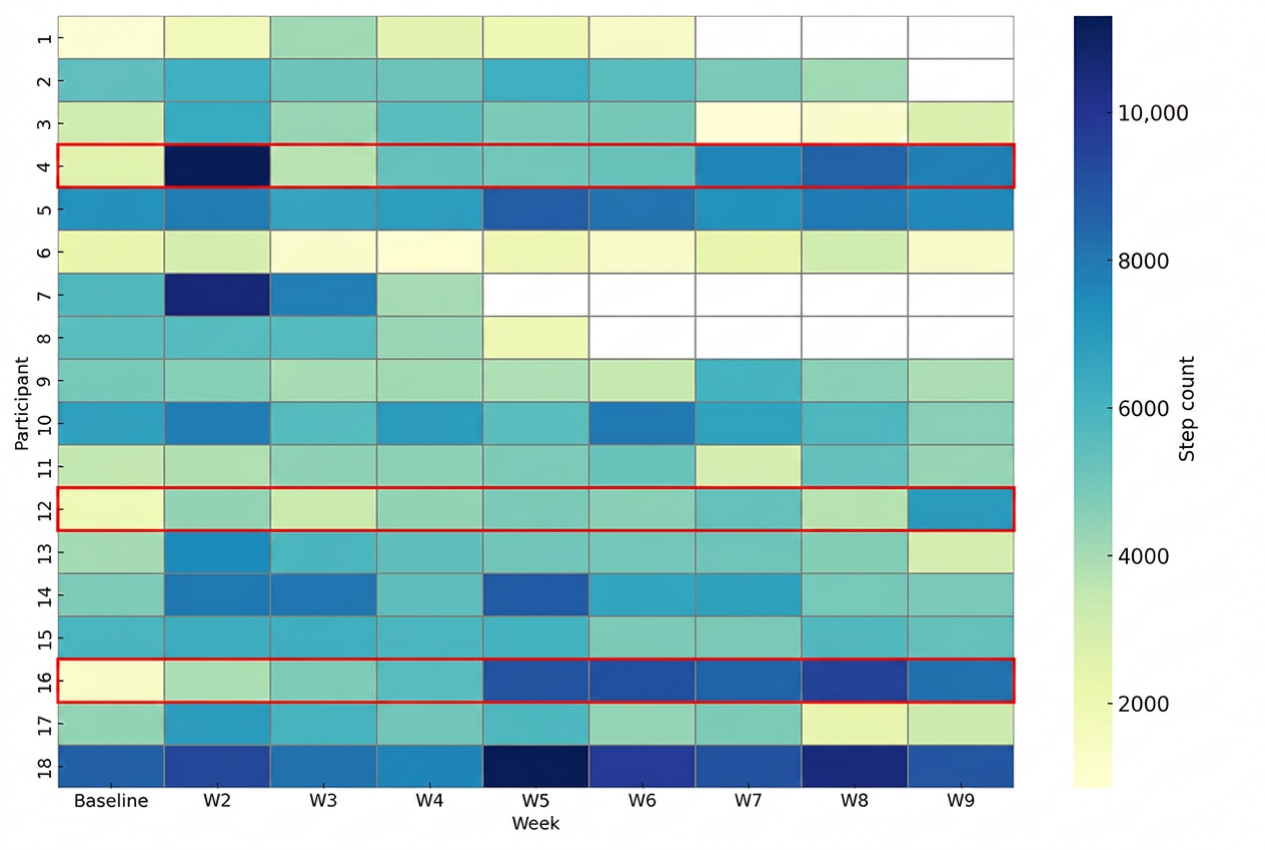
Heatmap of smartphone-based step counts over time among participants in a proof-of-concept and feasibility study of MoodMover for adults with depression. Each row represents 1 participant. Red borders indicate participants with >3000 daily steps per week increase at week 9.

Multimedia Appendix 8 presents changes in smartphone-based step counts and other self-reported measures from baseline to post intervention. Of the 21 participants, 18 successfully synced their step data to Pathverse, with a mean of 4378.4 (SD 2114.5) daily steps (n=18) recorded in week 1. By week 9, the average changed to 5204.0 (SD 2322.5) steps (n=14), with a median change of −131.5 steps. The CIs (75% CI −760.05 to 385.36, 85% CI −944.14 to 394.14, and 95% CI −947.50 to 859.57, respectively) suggest that this change was not statistically significant. Figure 3 displays a heatmap of each participant’s weekly smartphone-based step counts across the 9-week period. Out of 14 participants, 3 (21%) achieved an increase of at least 3000 steps over the 9 weeks. All 3 reported linking their smartwatches with the Health app (iOS) or Google Fit (Android), and 2 of them wore their smartwatches consistently on both weekdays and weekends. Among the remaining 11 participants who provided valid step data across all 9 weeks, 5 did not use smartwatches, and 3 reported seldom or only sometimes carrying their phones. Of the 6 who linked smartwatches, only 2 reported wearing them consistently.

In contrast to the step count data, participants self-reported engaging in a mean of 82.9 (SD 86.4) minutes of MVPA per week at baseline (n=28), which increased to 206.8 (SD 193.2) minutes (n=16) post intervention, with a median difference of 57.5 minutes (75% CI −17.50 to 170.00, 85% CI −25.00 to 185.00, and 95% CI −27.44 to 210.00, respectively). After excluding 1 extreme postintervention value (>3 SD from the mean), the mean was 167.3 (SD 114.8) minutes per week, yielding a mean difference of 71.3 minutes. The corresponding 75%, 85%, and 95% CIs were 28.10-114.43 minutes, 16.48-126.05 minutes, and −5.88 to 148.41 minutes, respectively.

Depression scores decreased from preintervention (mean 14.0, SD 4.4) to post intervention (mean 7.9, SD 5.7), with a mean difference of −5.31. The 75%, 85%, and 95% CIs were −7.04 to −3.59, −7.50 to −3.12, and −8.39 to −2.24, respectively, indicating a robust effect. Among 12 participants with baseline PHQ-9 scores of ≥10, 9 (75%) achieved a clinically meaningful reduction of ≥5 points, shifting from ≥10 at baseline to ≤9 post intervention. Among these 12 participants, the 2-tailed paired *t* test revealed a mean difference of −6.50. The 75%, 85%, and 95% CIs were −8.62 to −4.38, −9.20 to −3.80, and −10.34 to −2.66, respectively.

All M-PAC constructs showed either significant or nonsignificant increases at postintervention, except for intention strength and decisional intention. Intention strength showed a small, nonsignificant decrease (mean difference=−0.08), with all CIs crossing zero. Decisional intention demonstrated a nonsignificant decrease in the likelihood of endorsing “Yes” post intervention (odds ratio 0.33), with all CIs crossing 1 (75% CI 0.08-1.06, 85% CI 0.06-1.30, and 95% CI 0.03-1.86, respectively). Regulation exhibited the largest and statistically significant improvement, with a mean difference of 0.81. The 75%, 85%, and 95% CIs were 0.54-1.08, 0.47-1.15, and 0.34-1.29, respectively.

### Correlations Between Changes in PA and Depression

A Pearson correlation indicated a moderate negative association between changes in smartphone-based step count and depression (*r*=−0.55; 75% CI −0.75 to −0.27, 85% CI −0.78 to −0.18, and 95% CI −0.84 to −0.03, respectively). In contrast, a Spearman rank-order correlation, bootstrapped with 2000 resamples, indicated a weaker, nonsignificant association (ρ=−0.29; 75% CI −0.59 to 0.06, 85% CI −0.66 to 0.15, and 95% CI −0.76 to 0.31, respectively).

Additionally, Pearson correlation revealed a strong negative association between changes in self-reported MVPA and depression (*r*=−0.77; 75% CI −0.87 to −0.60, 85% CI −0.89 to −0.54, and 95% CI −0.91 to −0.44, respectively). Similarly, Spearman rank correlation indicated a strong negative relationship (ρ=−0.85; 75% CI −0.91 to −0.73, 85% CI −0.93 to −0.69, and 95% CI −0.95 to −0.61, respectively), with bootstrapping based on 2000 resamples. After excluding the identified outlier, both Pearson and Spearman correlations indicated similarly strong negative associations, with coefficients of *r*=−0.80 and ρ=−0.82, respectively (Multimedia Appendix 9).

## Discussion

### Principal Findings

This proof-of-concept and feasibility study represents an early stage in the development of MoodMover, an app-based intervention designed to increase PA among individuals with depression. All 4 feasibility criteria fell within the amber zone, indicating suboptimal feasibility and the need for modifications. Despite this, self-reported MVPA increased substantially, and clinically meaningful improvements in depressive symptoms were observed among most study completers who had self-reported at least moderate levels of depressive symptoms. Although no significant changes were detected in smartphone-based step counts or several secondary outcomes, the study was not powered to detect such effects. Overall, these findings support cautious progression to the next subphase of the ORBIT model, phase IIc: Efficacy, to evaluate an upgraded version of MoodMover with the necessary amendments in a fully powered RCT.

### Feasibility and Acceptability

Our study demonstrated a recruitment rate of 63%, slightly below the predetermined target of 65%. This aligns with a previous digital health intervention with a mobile health (mHealth) app for depression and anxiety, which reported a recruitment rate of 63.2% among eligible participants [55]. Although the criterion was not met, recruitment remained promising, particularly given the relatively modest nature of the recruitment strategy, which largely relied on the REACH BC website. This approach limited exposure to individuals not registered on the platform, thereby reducing the broad accessibility typically offered by mHealth interventions. Limited engagement from advocacy groups and mental health associations further constrained access to individuals more likely to be interested in self-help programs. The limited enrollment from advocacy groups likely reflects insufficient upfront investment in building and maintaining these relationships, such as the absence of co-designed recruitment materials, rather than a lack of potential interest from these organizations or their constituents. Involving mental health partners early in the design and development process may improve reach and promote the scalability of the intervention. Future studies with a larger sample size could incorporate social media recruitment (eg, Facebook, X, and Reddit) and establish stronger partnerships with health care providers and mental health associations to enhance outreach and engage hard-to-reach populations [56]. Although still in early stages, we have initiated discussions with CANMAT to develop endorsement pathways, whereby clinicians could use CANMAT-endorsed materials to introduce MoodMover during routine consultations. Such partnerships could enhance credibility, reach, and scalability in future larger-scale trials.

A retention rate of 57% (16/28) was observed in this study. Although this fell within the amber zone of the predetermined progression criterion, it was either comparable to or better than the attrition rates reported in previous digital interventions for depression. A review by Richards and Richardson [57] reported a 57% attrition rate in computer-based psychological treatments for depression, with substantially higher dropout rates (74%) observed in unsupported interventions. More recently, Linardon and Fuller-Tyszkiewicz [58] found a mean attrition rate of 32.1% (95% CI 17.0%-52.2%) at long-term follow-up (>8 weeks) across smartphone-based interventions for depressive symptoms, although substantial heterogeneity was noted (*I*²=96%). Torous et al [59] found a lower pooled dropout rate of 26.2% but also reported considerable heterogeneity (*I*²=93.4%) and significant publication bias (Egger *t*=3.9, *P*<.001). When adjusted for publication bias, the estimated attrition rate increased to 47.8% (95% CI 35.8%-60.0%) [59]. Given that MoodMover provided no ongoing support beyond an initial orientation session and access to technical assistance, the observed retention rate appears promising. In addition, more than half of the attrition (7/12, 58%) occurred prior to the orientation session. Two participants cited an overwhelming enrollment process as their reason for withdrawal, suggesting that barriers unrelated to the intervention content may have contributed to early dropout. When excluding participants who did not receive any intervention, the retention rate increased to 76% (16/21). This figure aligns with retention rates observed in previous app-based interventions that included mood monitoring features, where dropout rates averaged 18.4% (95% CI 10.9%-29.4%) [59]. These findings highlight the importance of refining the MoodMover research protocol, particularly by simplifying the enrollment process to enhance participant retention. For instance, replacing text-based preparation guidance for orientation with a short instructional video could improve accessibility. While a comprehensive user guide can be informative, it may feel overwhelming for participants, especially those with more severe depressive symptoms. Embedding the guide within the app as an optional resource could reduce perceived burden while maintaining usability. More feasibility trials can be conducted to explore optimal enrollment and strategies to enhance retention before moving forward to a large RCT.

Similarly, the adherence rate of the current version of MoodMover (67%) fell within the amber zone of the pre-set criterion (40%‐70%), indicating that potential modifications may be needed before proceeding to an RCT. Although it did not reach the green zone, the adherence rate was higher than that of most previous mHealth apps for depression, which have reported low adherence rates (<50%) in prior reviews [58,60,61]. For trials targeting depression, Linardon and Fuller-Tyszkiewicz [58] found a mean percentage of “nonusers” of 41.2% (*k*=2; range 25%‐58%), a mean percentage of “complete adherers” of 34% (*k*=3; range 14%‐68%), and mean numbers of logins ranging from 14 to 21 times (*k*=3). In this study, no participants were classified as nonusers after completing the orientation session. Additionally, a relatively high rate of complete users was observed, with 47.6% (10/21) completing all major modules. This may be attributed to several strategies implemented to enhance adherence, such as gamification points with modest incentives for lesson completion, the involvement of end users in the development and design process prior to the launch of this trial, and the integration of a peer support community forum [62]. Nevertheless, despite usability testing conducted prior to the trial, more than half of the participants encountered different levels of unexpected technical problems during the program, which may have adversely affected their adherence and app engagement. This was reflected in the fact that only half of the participants reported an acceptable level of usability, and satisfaction ratings varied considerably. User-friendliness and technical stability have been identified as key factors influencing adherence to mHealth interventions [63]. The functionality of MoodMover should therefore be further refined and rigorously tested prior to a future trial. Additionally, future research should further explore and test strategies that may enhance adherence.

Promisingly, participants engaged with the app on an average of 30.8 days, suggesting relatively sustained engagement compared with typical patterns in health app usage in real-world settings [64]. Weekly app use duration declined over time, with sharper drops from weeks 2-3 and weeks 5-6, then appearing to stabilize between and after these periods. This declining usage trajectory over time is commonly observed in smartphone-based interventions [58]. The large standard deviations indicated substantial variability in engagement, with some participants using the app considerably more than others. This pattern was consistently reflected in the use of specific app features, which varied in both frequency and number of users. Step tracking, exercise logging, and mood tracking were the most commonly used features. In contrast, fewer participants engaged with the action planning and community forum components, and usage of these features was limited and inconsistent.

MoodMover included daily prompts to sync steps, log exercise sessions, and reflect on mood. These reminders aligned with the features that participants engaged with most frequently. This may suggest that tailoring notifications to specific app features may reinforce user engagement and support continued use of those components. Future studies could explore adaptive notification strategies and personalized messaging as tools for improving engagement and encouraging behavior change among individuals with depression. Moreover, total app use time and its variability across participants were likely underestimated due to the unavailability of data on time spent in features under the menu tab, particularly the Resources section, which includes exercise videos. The Pathverse platform could be further improved to provide more accurate and comprehensive metadata.

Regression analyses revealed that age was the only participant-level factor that consistently influenced app engagement, usability, and satisfaction in this study. This finding aligns with the observed trend that younger participants were more likely to drop out during the intervention. Due to limited information on reasons for early discontinuation in this study, we were unable to offer a proper explanation for this pattern. Future research should further investigate factors influencing engagement, implement systematic tracking of dropout reasons, and explore strategies specifically aimed at supporting sustained engagement, particularly among younger users.

### Behavioral and Clinical Outcomes

Only 3 participants achieved the behavioral goal of increasing their daily step count by 3000 steps per week, as measured by smartphone-generated data. A likely contributing factor was the inconsistent use of tracking devices across the intervention: most participants (7/11, 64%) who failed to achieve a 3000-step increase reported that they inconsistently carried their smartphone and/or inconsistent use of their smartwatch. Consequently, reported that step count data are not an accurate representation of actual daily steps. In addition, 56% (9/16) of the participants reported concerns related to the step syncing component, including perceived inaccuracy and the inconvenience of needing to carry their phones. This interpretation is supported by discrepancies with self-reported MVPA: participants reported an average increase of 57.5 minutes of MVPA per week at postintervention, which increased to 71.3 minutes per week after excluding 1 extreme outlier. The latter results were statistically significant at the 75% and 85% CIs, although not at the 95% CI. Future evaluation of MoodMover should include device-based PA measurement through accelerometry, with complementary devices (eg, Fitbits) to facilitate participant self-monitoring.

More promisingly, this study found a mean reduction of 5.31 points on the PHQ-9, with statistically significant results across all CIs. Among participants with moderate to severe depressive symptoms at baseline (PHQ-9 ≥10), 75% (9/12) reported a clinically meaningful reduction at postintervention, with a mean reduction of 6.50 points on the PHQ-9. The CIs excluded zero but included the predetermined threshold of 5, suggesting a potentially clinically meaningful effect [53]. In addition, improvements in PA levels, particularly self-reported MVPA, were moderately to strongly associated with reductions in depressive symptoms. In terms of M-PAC constructs, affective attitudes and regulations showed the largest significant increases, with mean changes of 0.65 and 0.81, respectively. The 2 reflexive constructs, habit and identity, demonstrated small increases, shifting from a level of disagreement to a more neutral stance. As the regulatory and reflexive constructs are critical for behavioral maintenance [23] and given the potential importance of perceived emotional benefits for individuals with depression [65], the favorable changes in PA and depression may be attributable in part to the intervention. While these findings were not definitive due to the small sample size, they are encouraging and support progression to the next phase of the ORBIT model, where a larger trial with sufficient power could be conducted to demonstrate statistical significance.

### Strengths and Limitations

A key strength of this study was the systematic development of MoodMover, guided by the M-PAC framework, following 2 established behavior change intervention development frameworks. This approach also enabled the integration of theory-driven BCTs. This study followed the BCTTv1 [32] to report the applied BCTs, including intended and unintended, in a detailed and transparent manner. Using standardized BCT definitions enhances the potential for future studies to synthesize evidence on the effectiveness of specific techniques in digital PA change interventions for depression. In addition, this study reported a range of objective app engagement data that provided valuable insights into the feasibility and acceptability of MoodMover. Given the multicomponent nature of most mHealth interventions, such data are essential for guiding future refinement and optimization of the app. Another strength of this study was the use of validated measurement tools, which enhances the reliability and comparability of the findings. In addition, the integration of device-based PA tracking via smartphones or smartwatches allowed for continuous monitoring of changes in activity levels throughout the intervention. However, this approach had an important limitation. Smartphone-based tracking depends on participants consistently carrying their phones, which likely resulted in an underestimation of actual activity levels.

Further limitations should also be noted. The generalizability of the findings is limited. All participants were required to own a smartphone, which may have excluded individuals from lower socioeconomic backgrounds. However, smartphone ownership in Canada is relatively high, with an estimated 84.4% of the population aged 15 years and older owning a smartphone as of 2020 [66]. In addition, while improvements in self-reported PA and reductions in depressive symptoms were observed, the small sample size limits the ability to draw firm conclusions about the efficacy of MoodMover. Moreover, as noted earlier, there was a deviation from the protocol regarding the fidelity checking process due to short intervals between orientation sessions. While this deviation was unlikely to have affected the feasibility, acceptability, or preliminary effects of MoodMover, a more practical fidelity checking procedure should be developed for future studies. This may involve scheduling orientation sessions with sufficient spacing between participants or incorporating automated, interactive instructions in Pathverse, which could ultimately eliminate the need for live orientations and reduce participant burden. Additionally, technical issues encountered during both the orientation sessions and the intervention phase may have significantly impacted the feasibility and proof-of-concept results. Furthermore, only 25% (7/28) of the participants identified as male, and just 2 male participants completed the study. This highlights the need to evaluate the feasibility and acceptability of MoodMover in a more diverse and representative population. Finally, the long-term maintenance of intervention effects remains unknown. Future trials should incorporate structured follow-up assessments to examine the sustainability of observed outcomes.

### Conclusions

Overall, the proof-of-concept findings are encouraging and support the potential of MoodMover in increasing PA among individuals with depression. However, the usability and feasibility of the current version were suboptimal, indicating that a larger-scale RCT should proceed only after necessary amendments have been implemented and evaluated. This suggests moving backward to the previous phase of the ORBIT model (phase Ib: Refine) to refine MoodMover. One or more feasibility studies assessing the upgraded version of MoodMover are recommended to further examine its usability and feasibility before progressing to a full-scale trial.

More broadly, this study underscores the value of phased intervention development, as exemplified by the ORBIT model, in ensuring that digital behavior change tools are both feasible and demonstrate proof-of-concept before proceeding to costly efficacy trials. The findings also highlight the importance of integrating device-based and subjective measures of PA in future research, as discrepancies between these metrics can reveal insights into both engagement patterns and measurement limitations. As smartphone-based interventions continue to proliferate, systematic refinement grounded in feasibility data will be essential to translating promising ideas into scalable, real-world supportive tools for individuals living with depression.

## Supplementary material

10.2196/79033 Multimedia Appendix 1 A list of unique behavior change techniques used in this version of MoodMover.

10.2196/79033 Multimedia Appendix 2 Questionnaires.

10.2196/79033 Multimedia Appendix 3 Baseline characteristics.

10.2196/79033 Multimedia Appendix 4 App engagement, usability, and satisfaction.

10.2196/79033 Multimedia Appendix 5 Smartphone-based step counts and app use over time.

10.2196/79033 Multimedia Appendix 6 Technical issues reported.

10.2196/79033 Multimedia Appendix 7 Simple linear regression results for participant characteristics of feasibility results.

10.2196/79033 Multimedia Appendix 8 Changes in smartphone-based step counts and other self-reported measures from baseline to postintervention.

10.2196/79033 Multimedia Appendix 9 Correlations between changes in physical activity and Patient Health Questionnaire, 9-Item.

10.2196/79033 Multimedia Appendix 10 ChatGPT transcripts.

10.2196/79033 Checklist 1 STROBE and CONSORT 2020 checklists.

## Abbreviations

BCT: behavior change technique
BCTTv1: BCT Taxonomy version 1
CANMAT: Canadian Network for Mood and Anxiety Treatments
CONSORT: Consolidated Standards of Reporting Trials
M-PAC: Multi-Process Action Control
MAUQ: mHealth app usability questionnaire
MDD: major depressive disorder
mHealth: mobile health
MVPA: moderate to vigorous physical activity
ORBIT: Obesity-Related Behavioural Intervention Trials
PA: physical activity
PHQ-9: Patient Health Questionnaire, 9-Item
RCT: randomized controlled trial
REDCap: Research Electronic Data Capture
STROBE: Strengthening the Reporting of Observational Studies in Epidemiology
