# Supplementary material for: App-Based Physical Activity Intervention for Individuals With Depression (MoodMover): Single-Arm, Pre-Post Proof-of-Concept and Feasibility Study
Source: JMIR Form Res. 2026 Jun 11;10:e79033. doi: 10.2196/79033 (PMC13256492; doi:10.2196/79033)
Supplement: Multimedia Appendix 1 [file formative-v10-e79033-s001.docx]

Multimedia Appendix 1. A list of unique BCTs used in this version of MoodMover.

| **No.** | **Behaviour Change Techniques^a^** | **Certainty** |
| --- | --- | --- |
| 1 | Goal setting (behaviour) (1.1) | ++ |
| 2 | Problem solving (1.2) | ++ |
| 3 | Action planning (1.4) | ++ |
| 4 | Review behaviour goal(s) (1.5) | + |
| 5 | Discrepancy between current behaviour and goal (1.6) | ++ |
| 6 | Self-monitoring of behaviour (2.3) | ++ |
| 7 | Self-monitoring of outcome(s) of behaviour (2.5) | ++ |
| 8 | Social support (unspecified) (3.1) | ++ |
| 9 | Social support (emotional) (3.3) | ++ |
| 10 | Instruction on how to perform a behaviour (4.1) | + |
| 11 | Information about antecedents (4.2) | ++ |
| 12 | Re-attribution (4.3) | ++ |
| 13 | Information about health consequences (5.1) | ++ |
| 14 | Monitoring of emotional consequences (5.4) | ++ |
| 15 | Information about emotional consequences (5.6) | ++ |
| 16 | Information about others' approval (6.3) | ++ |
| 17 | Prompts/cues (7.1) | ++ |
| 18 | Behaviour substitution (8.2) | ++ |
| 19 | Habit formation (8.3) | ++ |
| 20 | Graded tasks (8.7) | ++ |
| 21 | Credible source (9.1) | ++ |
| 22 | Social reward (10.4) | + |
| 23 | Non-specific incentive (10.6) | ++ |
| 24 | Self-incentive (10.7) | ++ |
| 25 | Reduce negative emotions (11.2) | + |
| 26 | Restructuring the physical environment (12.1) | ++ |
| 27 | Restructuring the social environment (12.2) | + |
| 28 | Identification of self as role model (13.1) | + |
| 29 | Incompatible beliefs (13.3) | + |
| 30 | Verbal persuasion about capability (15.1) | + |
| 31 | Self-talk (15.4) | ++ |

^a^The numbers in the brackets refer to the behaviour change techniques in the ‘BCT taxonomy v1’ by Michie et al. 2013.
