# Supplementary material for: App-Based Physical Activity Intervention for Individuals With Depression (MoodMover): Single-Arm, Pre-Post Proof-of-Concept and Feasibility Study
Source: JMIR Form Res. 2026 Jun 11;10:e79033. doi: 10.2196/79033 (PMC13256492; doi:10.2196/79033)
Supplement: Multimedia Appendix 2 [file formative-v10-e79033-s002.docx]

**Multimedia Appendix 2. Questionnaires.**

**1. Screening questionnaire.**

Chapter 1.

1. Are you aged between 18-64 years?

- Yes
- No (if selected, proceed to End of survey)

2. Do you have a smartphone, either iPhone or Android, with internet access that can download and use an app?

- Yes
- No (if selected, proceed to End of survey)

3. Are you able to read English? Are you able to respond to questions in spoken English?

- Yes
- No (if selected, proceed to End of survey)

4. Do you currently have a clinical diagnosis of a major depressive disorder?

- Yes
- No

5. If any, do you anticipate any major changes to your current antidepressant treatment regimen (e.g., changes in medication type or dosage, adjustments to psychotherapy) within the next 3 months?

- Yes (if selected, proceed to End of survey)
- No
- I’m not taking any antidepressant treatments

6. Do you currently have any physical disabilities that prevent you from engaging in regular exercise or physical activity?

- Yes (if selected, proceed to End of survey)
- No

7. Have you experienced any of the following conditions in the past month: active psychosis or mania, active suicidal thoughts, or severe difficulties with thinking and memory?

- Yes (if selected, proceed to End of survey)
- No

8. Do you have an active alcohol or substance use disorder?

- Yes (if selected, proceed to End of survey)
- No

9. If you are female, are you currently pregnant?

- Yes (if selected, proceed to End of survey)
- No
- Not applicable

10. Have you been diagnosed with another major primary psychiatric disorder, such as psychosis?

- Yes (if selected, proceed to End of survey)
- No

10. In the past week, on how many days have you done a total of 30 minutes or more of physical activity, which was enough to raise your breathing rate? This may include sport, traditional games, exercise, and brisk walking or cycling for recreation or to get to and from places, but should not include housework or physical activity that may be part of your job.

- 0
- 1
- 2
- 3
- 4
- 5
- 6
- 7

If >= 3 days/per week (achieving 90 mins of moderate-to-vigorous physical activity per week), procced to End of survey.

11. Do you anticipate a major absence (e.g., monthly long vacation, surgery) in the next 3 months?

- Yes (if selected, proceed to End of survey)
- No

12. Do you have a valid and active email address that you prefer to use to communicate with us and engage in this study?

- Yes (if selected, proceed to Q13)
- No (if selected, proceed to End of survey)

13. Please provide your email address:

Chapter 2: Patient Health Questionnaire, 9-item (PHQ-9)

| Over the last 2 weeks, how often have you been bothered by any of the following problems? | Not at all | Several days | More  than half the days | Nearly every day |
| --- | --- | --- | --- | --- |
| 1. Little interest or pleasure in doing things | 0 | 1 | 2 | 3 |
| 2. Feeling down, depressed, or hopeless | 0 | 1 | 2 | 3 |
| 3. Trouble falling or staying asleep, or sleeping too much | 0 | 1 | 2 | 3 |
| 4. Feeling tired or having little energy | 0 | 1 | 2 | 3 |
| 5. Poor appetite or overeating | 0 | 1 | 2 | 3 |
| 6. Feeling bad about yourself – or that you are a failure or have let yourself or your family down | 0 | 1 | 2 | 3 |
| 7. Trouble concentrating on things, such as reading the newspaper or watching television | 0 | 1 | 2 | 3 |
| 8. Moving or speaking so slowly that other people could have noticed? Or the opposite – being so fidgety or restless that you have been moving around a lot more than usual | 0 | 1 | 2 | 3 |
| 9. Thoughts that you would be better off dead or of hurting yourself in some way | 0 | 1 | 2 | 3 |

1. How difficult have these problems made it for you to do your work, take care of things at home, or get along with other people?

- Not difficult at all
- Somewhat difficult
- Very difficult
- Extremely difficult

**2.** **Demographic survey and clinical information.**

1. What is your age?
2. What is your height (m)?
3. What is your weight (kg)?
4. Please indicate your sex at birth.

- Female
- Male
- Prefer not to Answer

1. Please indicate your gender.

- Woman
- Man
- Non-binary person
- Prefer not to Answer

1. The Canadian Census identifies the following categories in its Census of the Population. Please indicate how you self-identify. This self-identification is not intended as an indication of one’s place of origin, citizenship, language or culture and recognizes that there are differences both between and among subgroups of persons of colour. If you are of mixed-descent, please indicate this by selecting all that apply, rather than using the “other” line unless parts of your self-identification do not appear in this list.

- Indigenous peoples of Canada
- Indigenous (outside of Canada)
- Arab
- Black
- Chinese (including Mainland China, Hong Kong, Macau and Taiwan)
- Filipino
- Japanese
- Korean
- Latin, Central, or South American (e.g., Brazilian, Chilean, Colombian, Mexican)
- South Asian (e.g., Indian, Pakistani, Sri Lankan, etc.)
- Southeast Asian (e.g., Cambodian, Indonesian, Laotian Vietnamese, etc.)
- West Asian (e.g., Afghan, Iranian, Syrian, etc.)
- White
- Other
- Prefer not to answer

1. Please indicate your ancestry here (if “Other” is selected to Q3).
2. What is the highest degree or level of education you have completed?

- Some high school
- High school
- Certificate or diploma
- Bachelor’s degree
- Master’s degree
- PhD
- Professional degree
- Prefer not to answer

1. What is your current employment status?

- Employed – part time (including self-employed)
- Employed – full time (including self-employed
- Stay-at-home parent
- Homemaker
- On maternity/parental leave
- On medical or disability
- Retired
- Unemployed
- Other
- Prefer not to answer

1. Please indicate your current employment status here (if “Other” is selected to Q6).
2. What is your before-tax household income?

- $0 - $19,999
- $20,000 - $39,999
- $40,000 - $59,999
- $60,000 - $79,999
- $80,000 - $99,999
- $100,000 - $119,999
- $120,000 - $139,999
- $140,000 or greater
- Prefer not to answer

1. Do you currently have a clinical diagnosis of major depressive disorder?

- Yes 🡪 13
- No 🡪 14

1. How long have you been diagnosed with depression?

- Less than 1 month
- 1-6 months
- 6-12 months
- 1-2 years
- More than 2 years

1. How long have you been experiencing symptoms of depression?

- Less than 1 month
- 1-6 months
- 6-12 months
- 1-2 years
- More than 2 years

1. Are you currently taking any medication for depression?

- Yes 🡪 16
- No 🡪 18

1. Please specify the name(s) and dosage(s) of the medication(s) you are currently taking:
2. How long have you been taking this/these medication(s)?

- Less than 1 month
- 1-6 months
- 6-12 months
- 1-2 years
- More than 2 years

1. Are you currently undergoing any other treatments for depression (e.g., psychotherapy, counseling, etc.)?

- Yes 🡪 19
- No 🡪 26

1. If yes, please specify the type(s) of treatment(s):
2. How long have you been receiving this/these treatment(s)?

- Less than 1 month
- 1-6 months
- 6-12 months
- 1-2 years
- More than 2 years

1. On a scale of 1 to 10, how effective do you find your current treatment for managing your depression symptoms? Scale from 1 (Not effective) to 10 (Very effective)
2. Have you noticed any side effects from your current treatment?

- Yes
- No

1. If yes, please specify the side effects:
2. Do you have any other ongoing medical conditions that are being treated along with your depression?

- Yes
- No

1. If yes, please specify the condition(s) and treatment(s):
2. Have you previously been treated for depression?

- Yes 🡪 25
- No 🡪 26

1. If yes, please specify the type(s) of previous treatment(s) and duration:

**Alcohol Use (From CCWS)**

For the purpose of this survey, a drink means:

• 341 ml or 12 oz. of beer or cooler (bottle, can, or draft)

• 142 ml or 5 oz. of wine

• 43 ml or 1.5 oz. of liquor or spirit (straight or mixed)

Include light beer.

Exclude de-alcoholised beer or coolers (0.5% alcohol) or cocktails such as Virgin Mary or Shirley Temple.

The physiological effects of alcohol vary by biological sex. For this question, refer to the number of drinks that corresponds with your biological sex at birth.

1. During the past 30 days, how often have you had 4 or more drinks (female sex) OR 5 or more drinks (male sex) on one occasion? *“On one occasion” means at the same time or within a couple of hours of each other.*

- Daily or almost daily
- 2 to 5 times a week
- Once a week
- 2 to 3 times in the past 30 days
- Once in the past 30 days
- Not in the past 30 days
- I do not drink alcohol
- I don't know
- I prefer not to answer

**Cannabis Use (From CCWS)**

The next questions are about **cannabis**. In this survey when we use the term cannabis, this includes **marijuana (e.g., weed, pot), hashish, hash oil or any other products made from the cannabis plant, but not synthetic cannabinoids.**

When we ask about use, this includes using cannabis in its dry form or when mixed or processed into another product such as an edible, an extract, a concentrate, including hashish, a liquid, or other product.

Cannabis use may include use for medical and/or non-medical purposes.

1. In the past 12 months, have you used cannabis?

- Yes 🡪 30
- No
- I don’t know
- I prefer not to answer

1. If yes, in the past 30 days, how often did you use cannabis?

- Not in the past 30 days
- 1 day in the past 30 days
- 2 or 3 days in the past 30 days
- 1 or 2 day(s) per week
- 3 or 4 days per week
- 5 or 6 days per week
- Daily
- I don't know
- I prefer not to answer

1. Are you currently using any other physical activity apps or devices (e.g., Fitbit, Garmin, Apple Watch)?

o Yes 🡪 32

o No 🡪 33

1. How frequently do you use these physical activity apps or devices?

o Not in the past 30 days

o 1 day in the past 30 days

o 2 or 3 days in the past 30 days

o 1 or 2 day(s) per week

o 3 or 4 days per week

o 5 or 6 days per week

o Daily

o I don't know

o I prefer not to answer

1. Have you ever used any physical activity apps or devices in the past?

o Yes 🡪 34

o No

1. For how long did you use these physical activity apps or devices?

- Less than 1 week
- 2-4 weeks
- 1-2 months
- 2-3 months
- 3-4 months
- 4-6 months
- Over 6 months

**3. Adapted MAUQ – Patient version for standalone apps**

(Responses on a 1 to 7, Strongly Disagree to Strongly Agree, scale)

**Ease of use**

S1. The app was easy to use.

S2. It was easy for me to learn to use the app.

S3. The navigation was consistent when moving between screens.

S4. The interface of the app allowed me to use all the functions (such as setting a step goal, logging a physical activity session, receiving notifications) offered by the app.

**Interface and satisfaction**

S5. Whenever I made a mistake using the app, I could recover easily and quickly.

S6. I like the interface of the app.

S7. The information in the app was well organized, so I could easily find the information I needed.

S8. The app adequately acknowledged and provided information to let me know the progress of my action.

S9. I feel comfortable using this app in social settings.

S10. The amount of time involved in using this app has been fitting for me.

S11. I would use this app again.

S12. Overall, I am satisfied with this app.

**Usefulness**

S13. The app would be useful for my mental health and well-being.

**4. Melin’s mHealth Satisfaction Questionnaire**

1. **The mHealth Satisfaction Questionnaire, 14-item**

|  | ***Strongly Disagree***  *1* | *2* | *3* | *4* | ***Strongly Agree***  *5* |
| --- | --- | --- | --- | --- | --- |
| **What did you think about using the health app?** | | | | | |
| It was easy to use |  |  |  |  |  |
| It was good to use |  |  |  |  |  |
| The time spent using it has been acceptable |  |  |  |  |  |
| It has been difficult to remember to use it |  |  |  |  |  |
| The introduction of how to use it was sufficient |  |  |  |  |  |
| It was too time consuming |  |  |  |  |  |
| It interrupted me in my daily activities |  |  |  |  |  |
| It was boring to use |  |  |  |  |  |
| It was a disturbance |  |  |  |  |  |
| I can recommend it to others |  |  |  |  |  |
| **How did you experience the health app?** | | | | | |
| It has motivated me to change my lifestyle habits |  |  |  |  |  |
| It has helped me to understand the benefits of improving my lifestyle habits |  |  |  |  |  |
| It has helped me to understand how I need to change my lifestyle habits |  |  |  |  |  |
| It has helped me set personal goals for my lifestyle habits in a way that I could not have done on my own |  |  |  |  |  |

1. **Please list three aspects of the program/MoodMover that you particularly liked and three aspects that you disliked. You may refer to things such as the content, delivery, or support.**

**5. Physical Activity Adult Questionnaire (PAAQ)**

Assuming the interview is conducted on a **Monday**.

Answer categories are provided in parentheses.

The following questions are about various types of physical activities done in the last 7 days.

1. In the last 7 days, that is from last Sunday to yesterday, did you use active ways like walking or cycling to get to places such as work, school, the bus stop, the shopping centre or to visit friends (active transportation)?

(Yes or No)

2. In the last 7 days, on which days did you do these activities?

(Monday through Sunday)

3. How much time in total, in the last 7 days, did you spend doing these activities?

(Type in answer)

4. In the last 7 days, did you do sports, fitness or recreational physical activities, organized or non-organized? Examples are walking, home or gym exercise, swimming, cycling, running, skiing, dancing and all team sports.

(Yes or No)

5. Did any of these recreational physical activities make you sweat at least a little and breathe harder?

(Yes or No)

6. In the last 7 days, on which days did you do these recreational activities that made you sweat at least a little and breathe harder?

(Monday through Sunday)

7. In the last 7 days, how much time in total did you spend doing these activities that made you sweat at least a little and breathe harder?

(Type in answer)

8. In the last 7 days, did you do any other physical activities while at work, in or around your home or while volunteering? Examples are carrying heavy loads, shoveling, and household chores such as vacuuming or washing windows. (Yes or No)

9. Did any of these other physical activities make you sweat at least a little and breathe harder?

(Yes or No)

10. In the last 7 days, on which days did you do these other activities that made you sweat at least a little and breathe harder?

(Monday through Sunday)

11. In the last 7 days, how much time in total did you spend doing these activities that made you sweat at least a little and breathe harder?

(Type in answer)

12. If you have reported any minutes of physical activity that made you sweat at least a little and breathe harder. Of these activities, were there any of vigorous intensity, meaning they caused you to be out of breath?

(Yes or No)

13. In the last 7 days, how much time **in total** did you spend doing vigorous activities that caused you to be out of breath?

(Type in answer)

14. During the program, did you link a smartwatch or fitness device to the Health app (iOS) or Google Fit (Android)? **–[post-intervention only]**

(Yes or No)

15. How often did you carry your phone or wear your smartwatch during your non-sedentary waking hours on workdays? **–[post-intervention only]**

(Almost always, Sometimes, Seldom)

16. How often did you carry your phone or wear your smartwatch during your non-sedentary waking hours on weekends? **–[post-intervention only]**

(Almost always, Sometimes, Seldom)

**6.** **GAD-7 Anxiety**

| Over the last two weeks, how often have you been bothered by the following problems? | Not  at all | Several days | More  than half the days | Nearly every day |
| --- | --- | --- | --- | --- |
| 1. Feeling nervous, anxious, or on edge | 0 | 1 | 2 | 3 |
| 2. Not being able to stop or control worrying | 0 | 1 | 2 | 3 |
| 3. Worrying too much about different things | 0 | 1 | 2 | 3 |
| 4. Trouble relaxing | 0 | 1 | 2 | 3 |
| 5. Being so restless that it is hard to sit still | 0 | 1 | 2 | 3 |
| 6. Becoming easily annoyed or irritable | 0 | 1 | 2 | 3 |
| 7. Feeling afraid, as if something awful might happen | 0 | 1 | 2 | 3 |

Column totals _____ + _____ + _____ + _____ =

*Total score* **_______**

If you checked any problems, how difficult have they made it for you to do your work, take care of things at home, or get along with other people?

Not difficult at all Somewhat difficult Very difficult Extremely difficult

□ □ □ □

Source: Primary Care Evaluation of Mental Disorders Patient Health Questionnaire (PRIME-MD-PHQ). The PHQ was developed by Drs. Robert L. Spitzer, Janet B.W. Williams, Kurt Kroenke, and colleagues. For research information, contact Dr. Spitzer at ris8@columbia.edu. PRIME-MD® is a trademark of Pfizer Inc. Copyright© 1999 Pfizer Inc. All rights reserved. Reproduced with permission

Scoring GAD-7 Anxiety Severity

This is calculated by assigning scores of 0, 1, 2, and 3 to the response categories, respectively, of “not at all,” “several days,” “more than half the days,” and “nearly every day.” GAD-7 total score for the seven items ranges from 0 to 21.

0–4: minimal anxiety

5–9: mild anxiety

10–14: moderate anxiety

15–21: severe anxiety

**7. The Pittsburgh Sleep Quality Index (PSQI)**

Instructions: The following questions relate to your usual sleep habits during **the past month only**. Your answers indicate the most accurate reply for the **majority** of days and nights in the past month. **Please answer all the questions.**

During the past month,

1. When have you usually gone to bed? _________________

2. How long (in minutes) has it taken you to fall asleep each night? ________________

3. When have you usually gotten up in the morning? _________________

4. How many hours of actual sleep do you get at night? (This may be different than the numbers of hours you spend in bed) _________________

| 5. During the past month, how often have you had trouble sleeping because you… | Not during the past month (0) | Less than once a week (1) | Once or twice a week (2) | Three or more times a week (3) |
| --- | --- | --- | --- | --- |
| 1. Cannot get to sleep within 30 minutes |  |  |  |  |
| 1. Wake up in the middle of the night or early morning |  |  |  |  |
| 1. Have to get up to use the bathroom |  |  |  |  |
| 1. Cannot breathe comfortably |  |  |  |  |
| 1. Cough or snore loudly |  |  |  |  |
| 1. Feel too cold |  |  |  |  |
| 1. Feel too hot |  |  |  |  |
| 1. Have bad dreams |  |  |  |  |
| 1. Have pain |  |  |  |  |
| 1. Other reason(s), please describe, including how often you have had trouble sleeping because of this reason(s): |  |  |  |  |
| 6. During the past month, how often have you taken medicine (prescribed or “over the counter”) to help you sleep? |  |  |  |  |
| 7. During the past month, how often have you had trouble staying awake while driving, eating meals, or engaging in social activity? |  |  |  |  |
| 8. During the past month, how much of a problem has it been for you to keep up enthusiasm to get things done? |  |  |  |  |
|  | Very good (0) | Fairly good (1) | Fairly bad (2) | Very bad (3) |
| 9. During the past month, how would you rate your sleep quality overall? |  |  |  |  |

**Scoring the PSQI**

In scoring the PSQI, seven component scores are derived, each scored 0 (no difficulty) to 3 (severe difficulty). The component scores are summed to produce a global score (range 0 to 21). Higher scores indicate worse sleep quality.

**Component 1:** **Subjective sleep quality—question 9**

#9 Score **. . . . . . . . . . . . . . . . . . . . . . . . . . . . . . . . . . . . . . . . . . . . . . . . . . . .** C1______

**Component 2: Sleep latency—questions 2 and 5a**

#2 Score (≤15min=0; 16-30 min=1; 31-60 min=2; >60 min=3) +#5a Score

(if sum is equal 0=0; 1-2=1; 3-4=2; 5-6=3**) . . . . . . . . . . . . . . . . . . . . . . . . .** C2______

**Component 3: Sleep duration—question 4**

#4 Score (>7=0; 6-7=1; 5-6=2; <5=3**) . . . . . . . . . . . . . . . . . . . . . . . . . . . . .** C3______

**Component 4: Sleep efficiency—questions 1, 3, and 4**

(total # of hours asleep)/(total # of hours in bed) x 100

>85%=0, 75%-84%=1, 65%-74%=2, <65%=3 **. . . . . . . . . . . . . . . . . . . . . .**  C4______

**Component 5: Sleep disturbance—questions 5b-5j**

Sum of scores #5b to #5j (0=0; 1-9=1; 10-18=2; 19-27=3**) . . . . . . . . . . . . .** C5______

**Component 6: Use of sleep medication—question 6**

#6 Score **. . . . . . . . . . . . . . . . . . . . . . . . . . . . . . . . . . . . . . . . . . . . . . . . . . . .** C6______

**Component 7: Daytime dysfunction**

#7 Score + #8 Score (0=0; 1-2=1; 3-4=2; 5-6=3) **. . . . . . . . . . . . . . . . . . . . .** C7______

Add the seven component scores together______ **Global PSQI Score**_______

Citation: Buysse, DJ, Reynolds CF, Monk TH, Berman SR, Kupfer DJ: The Pittsburgh

Sleep Quality Index (PSQI): A new instrument for psychiatric research and

practice. Psychiatry Research 28:193-213, 1989

**8. WHODAS 2.0 12-item version, self-administered**

This questionnaire asks about difficulties due to health conditions. Health conditions include diseases or illnesses, other health problems that may be short or long lasting, injuries, mental or emotional problems, and problems with alcohol or drugs.

Think back over the past 30 days and answer these questions, thinking about how much difficulty you had doing the following activities. For each question, please circle only one response.

| In the past 30 days, how much difficulty did you have in: | | | | | | |
| --- | --- | --- | --- | --- | --- | --- |
| S1 | Standing for long periods such as 30 minutes? | None | Mild | Moderate | Severe | Extreme or cannot do |
| S2 | Taking care of your household responsibilities? | None | Mild | Moderate | Severe | Extreme or cannot do |
| S3 | Learning a new task, for example, learning how to get to a new place? | None | Mild | Moderate | Severe | Extreme or cannot do |
| S4 | How much of a problem did you have joining in community activities (for example, festivities, religious or other activities) in the same way as anyone else can? | None | Mild | Moderate | Severe | Extreme or cannot do |
| S5 | How much have you been emotionally affected by your health problems? | None | Mild | Moderate | Severe | Extreme or cannot do |

***Please continue to next page...***

| In the past 30 days, how much difficulty did you have in: | | | | | | |
| --- | --- | --- | --- | --- | --- | --- |
| S6 | Concentrating on doing something for ten minutes? | None | Mild | Moderate | Severe | Extreme or cannot do |
| S7 | Walking a long distance such as a kilometre [or equivalent]? | None | Mild | Moderate | Severe | Extreme or cannot do |
| S8 | Washing your whole body? | None | Mild | Moderate | Severe | Extreme or cannot do |
| S9 | Getting dressed? | None | Mild | Moderate | Severe | Extreme or cannot do |
| S10 | Dealing with people you do not know? | None | Mild | Moderate | Severe | Extreme or cannot do |
| S11 | Maintaining a friendship? | None | Mild | Moderate | Severe | Extreme or cannot do |
| S12 | Your day-to-day work? | None | Mild | Moderate | Severe | Extreme or cannot do |

| H1 | Overall, in the past 30 days, how many days were these difficulties present? | ***Record number of days*** |
| --- | --- | --- |
| H2 | In the past 30 days, for how many days were you totally unable to carry out your usual activities or work because of any health condition? | ***Record number of days*** |
| H3 | In the past 30 days, not counting the days that you were totally unable, for how many days did you cut back or reduce your usual activities or work because of any health condition? | ***Record number of days*** |

This completes the questionnaire. Thank you.

**9. M-PAC constructs**

**Physical Activity**

The next questions ask about your thoughts and feelings about physical activity. Before we begin, please take an extra minute to read this section. It is very important that you understand what we mean when we say ***physical activity.***

***Physical activity*** is movement that takes effort and makes your heart beat faster. You might start to breathe harder, feel warm or flushed, or sweat.

Examples of physical activity include brisk walking, swimming, tennis, cycling, basketball, hiking, or paddle boarding

**ATTITUDES TOWARD PHYSICAL ACTIVITY**

***Affective Attitudes*** (Rhodes et al., 2010)

For me, participating in regular physical activity over the next month would be:

*(Responses on a 1 to 7 scale)*

1. Unenjoyable to enjoyable
2. Boring to very exciting
3. Unpleasant to very pleasant

***Instrumental Attitudes*** (Rhodes et al., 2010)

For me, participating in regular physical activity over the next month would be:

*(Responses on a 1 to 7 scale)*

1. Unwise to very wise
2. Not beneficial to very beneficial
3. Useless to very useful

**PERCEIVED CAPABILITY** (Rhodes et al., 2006)

These questions ask you about your confidence and/or control over engaging in regular physical activity. Please select a number for each question using the scale provided.

Consider what you would do for each question, assuming you wanted to do the task.

Please indicate your level of agreement with the following statements:

*(Responses on a 1 to 5, Strongly Disagree to Strongly Agree, scale)*

1. I have the skills I need to be physically active
2. I am physically capable of doing moderate to vigorous physical activity for 150 min across a week if I really had to
3. I am confident in my ability to engage in physical activity.

**PERCEIVED OPPORTUNITY FOR PHYSICAL ACTIVITY** (Rhodes et al, 2006)

These questions ask you about your opportunity for engaging in regular exercise. Please circle a number for each question using the scale provided.

*(Responses on a 1 to 5, Strongly Disagree to Strongly Agree, scale)*

1. I have the opportunity to be physically active every week if I really had to
2. I will have opportunities to increase my physical activity over the next month
3. I will have opportunities to track my physical activities via the app over the next month.

**INTENTION STRENGTH TO BE PHYSICALLY ACTIVE** (Rhodes et al., 2010)

These questions ask you about your intention for engaging in regular exercise. Please circle a number for each question using the scale provided.

*(Responses on a 1 to 5, Strongly Disagree to Strongly Agree, scale)*

1. I am committed to engage in physical activity over the next month
2. I am motivated to engage in PA over the next month
3. In the next 6 months, I have intentions to be physically active regularly

**DECISIONAL INTENTIONS TO BE PHYSICALLY ACTIVE**

The following will ask you about your intention to be regularly physically active.

I intend to increase 3000 steps above my baseline daily steps for most days per week. (Yes/No)

**BEHAVIOURAL REGULATION FOR PHYSICAL ACTIVITY** (Rovniak et al. 2002)

Sometimes we use strategies to help us to be physically active. Please use the following scale to answer the questions below.

*(Responses on a 1 to 5, Strongly Disagree to Strongly Agree, scale)*

1. I often monitor how physically active I am
2. I often set physical activity goals
3. I often plan, when, what, where, and how I am going to be physically active

**HABIT OF PHYSICAL ACTIVITY** (Gardner, Abraham, Lally, & de Bruijn, 2012; Rhodes and Lim, 2016)

The following questions ask about your habits regarding physical activity. Habits are behaviors that are so practiced they are often done without much thought or motivation. For example, for many people driving a car becomes a habit when one can think about all sorts of things, concentrate on the road, and give little thought to operating the controls of the car. Sometimes travelling to and from work becomes such a habit that we do not even think about our route anymore. Please select the answer that best represents you.

*(Responses on a 1 to 5, Strongly Disagree to Strongly Agree, scale)*

1. I engage in regular physical activity without having to consciously remember it
2. I engage in regular physical activity automatically
3. I engage in regular physical activity without consciously thinking about it

**PHYSICAL ACTIVITY IDENTITY** (Wilson & Muon, 2008; Rhodes and Lim, 2016)

The following questions concern your personal beliefs about exercise. Please indicate the degree to which you agree or disagree with each statement when thinking about your exercise participation.

*(Responses on a 1 to 5, Strongly Disagree to Strongly Agree, scale)*

1. I consider myself someone who is physically active
2. When I describe myself to others, I usually include my involvement in physical activity
3. Others see me as someone who does physical activity regularly
