# Supplementary material for: App-Based Physical Activity Intervention for Individuals With Depression (MoodMover): Single-Arm, Pre-Post Proof-of-Concept and Feasibility Study
Source: JMIR Form Res. 2026 Jun 11;10:e79033. doi: 10.2196/79033 (PMC13256492; doi:10.2196/79033)
Supplement: Multimedia Appendix 3 [file formative-v10-e79033-s003.docx]

Multimedia Appendix 3. Baseline characteristics.

| **Variable** | **Total (N=28), n (%) / Mean ± SD** | **Completers (n=16), n (%) / Mean ± SD** | **Non-completers (n=12), n (%) / Mean ± SD** |
| --- | --- | --- | --- |
| **Age (years)** | 39.8 ± 13.4 | 44.1 ± 13.2 | 34.0 ± 12.0 |
| **BMI**^a^ **(kg/m²)** | 27.8 ± 5.1 | 26.9 ± 4.1 | 29.1 ± 6.2 |
| **Sex** |  |  |  |
| Male | 7 (25.0%) | 2 (12.5%) | 5 (41.7%) |
| Female | 21 (75.0%) | 14 (87.5%) | 7 (58.3%) |
| **Gender** |  |  |  |
| Men | 4 (14.3%) | 1 (6.2%) | 3 (25%) |
| Women | 20 (71.4%) | 13 (81.2%) | 7 (58.3%) |
| Non-binary | 3 (10.7%) | 2 (12.5%) | 1 (8.3%) |
| Prefer not to answer | 1 (3.6%) | 0 | 1 (8.3%) |
| **Race/Ethnicity** |  |  |  |
| White | 18 (64.3%) | 10 (62.5%) | 8 (66.7%) |
| Non-White | 10 (35.7%) | 6 (37.5%) | 4 (33.3%) |
| **Education Level** |  |  |  |
| High School or Less | 7 (25.0%) | 3 (18.8%) | 4 (33.3%) |
| Certificate or diploma | 7 (25.0%) | 4 (25.0%) | 3 (25.0%) |
| Bachelor’s Degree | 9 (32.1%) | 4 (25.0%) | 5 (41.7%) |
| Master’s Degree | 4 (14.3%) | 4 (25.0%) | 0 |
| Professional Degree | 1 (3.6%) | 1 (6.2%) | 0 |
| **Employment Status** |  |  |  |
| Employed Full-Time | 9 (32.1%) | 6 (37.5%) | 3 (25.0%) |
| Employed Part-Time | 3 (10.7%) | 2 (12.5%) | 1 (8.3%) |
| Student | 5 (17.9%) | 3 (18.8%) | 2 (16.7%) |
| On medical or disability | 7 (25.0%) | 4 (25%) | 3 (25%) |
| Unemployed/Other | 4 (14.3%) | 1 (6.2%) | 3 (25.0%) |
| **Before-Tax Household Income** |  |  |  |
| $ 0 - $ 19,999 | 9 (32.1%) | 4 (25.0%) | 5 (41.7%) |
| $20,000 - $39,999 | 5 (17.9%) | 1 (6.2%) | 4 (33.3%) |
| $40,000 - $59,999 | 1 (3.6%) | 1 (6.2%) | 0 |
| $60,000 - $79,999 | 2 (7.1%) | 2 (12.5%) | 0 |
| $80,000 - $99,999 | 1 (3.6%) | 0 | 1 (8.3%) |
| $100,000 - $119,999 | 1 (3.6%) | 0 | 1 (8.3%) |
| $120,000 - $139,999 | 2 (7.1%) | 2 (12.5%) | 0 |
| $140,000 or greater | 4 (14.3%) | 4 (25.0%) | 0 |
| Prefer not to answer | 3 (10.7%) | 2 (12.5%) | 1 (8.3%) |
| **PHQ-9**^b^ | 14.0 ± 4.4 | 13.2 ± 3.9 | 15.1 ± 4.9 |
| **MDD**^c^ **Diagnosis** |  |  |  |
| Yes | 17 (60.7%) | 9 (56.2%) | 8 (66.7%) |
| No | 11 (39.3%) | 7 (43.8%) | 4 (33.3%) |
| **Duration of MDD Diagnosis (n=17)** |  |  |  |
| 1-6 months | 1 (5.9%) | 1 (6.2%) | 0 |
| 6-12 months | 1 (5.9%) | 1 (6.2%) | 0 |
| 1-2 years | 2 (11.8%) | 1 (6.2%) | 1 (8.3%) |
| More than 2 years | 13 (76.5%) | 6 (37.5%) | 7 (58.3%) |
| **Duration of Depressive Symptoms, no MDD diagnosis (n=11)** | |  |  |
| 1-2 years | 1 (9.1%) |  | 1 (8.3%) |
| More than 2 years | 10 (90.9%) | 7 (43.8%) | 3 (25%) |
| **Currently on Antidepressants** | 24 (85.7%) | 14 (87.5%) | 10 (83.3%) |
| **Duration of Medication Use** |  |  |  |
| Less than 1 month | 1 (4.2%) | 1 (6.2%) | 0 |
| 1-6 months | 3 (12.5%) | 2 (12.5%) | 1 (8.3%) |
| 6-12 months | 4 (16.7%) | 1 (6.2%) | 3 (25%) |
| 1-2 years | 1 (4.2%) | 1 (6.2%) | 0 |
| More than 2 years | 14 (58.3%) | 9 (56.2%) | 5 (41.7%) |
| Prefer not to answer | 1 (4.2%) | 0 | 1 (4.2%) |
| **Currently in Other Treatments for Depression (n=13)** | 13 (56.5%) | 7 (43.8%) | 6 (50%) |
| Counselling | 10 (76.9%) | 5 (71.4%) | 5 (83.3%) |
| Group CBT^d^ | 1 (7.7%) | 1 (14.3%) | 0 |
| Other | 2 (15.4%) | 1 (14.3%) | 1 (16.7%) |
| **Duration of Such Treatments (n=13)** |  |  |  |
| 1-6 months | 2 (15.4%) | 1 (14.3%) | 1 (16.7%) |
| 6-12 months | 4 (30.8%) | 2 (28.6%) | 2 (33.3%) |
| 1-2 years | 2 (15.4%) | 1 (14.3%) | 1 (16.7%) |
| More than 2 years | 5 (38.5%) | 3 (42.9%) | 2 (33.3%) |
| **Effectiveness of Current Treatments (1-10 scale)** | 5.8 ± 1.7 (n = 23) | 5.7 ± 1.3 (n = 13) | 5.9 ± 2.1 (n = 10) |
| **Side Effects of Current Treatments** | 11 (37.8%) | 7 (63.6%) | 4 (36.4%) |
| **Currently in Other Treatments Not for Depression** | 13 (56.5%) | 10 (62.5%) | 3 (25.0%) |
| **Alcohol Use (past month)** |  |  |  |
| 2 to 5 times a week | 3 (10.7%) | 2 (12.5%) | 1 (8.3%) |
| 2 to 3 times in the past 30 days | 2 (7.1%) | 1 (6.2%) | 1 (8.3%) |
| Once in the past 30 days | 3 (10.7%) | 2 (12.5%) | 1 (8.3%) |
| Not in the past 30 days | 11 (39.3%) | 6 (37.5%) | 5 (41.7%) |
| I do not drink alcohol | 9 (32.1%) | 5 (31.2%) | 4 (33.3%) |
| **Cannabis Use (past 12 months)** |  |  |  |
| Yes | 10 (35.7%) | 6 (37.5%) | 4 (33.3%) |
| No | 18 (64.3%) | 10 (62.5%) | 8 (66.7%) |
| **Currently Using Other PA**^e^ **Apps or Devices** | 13 (46.4%) | 6 (37.5%) | 7 (58.3%) |
| Daily | 7 (53.8%) | 3 (50.0%) | 4 (57.1%) |
| Others | 6 (46.2%) | 3 (50.0%) | 3 (42.9%) |
| **History of Using Other PA Apps or Devices** |  |  |  |
| Yes | 10 (66.7%) | 8 (50%) | 2 (16.7%) |
| No | 5 (33.3%) | 2 (12.5%) | 3 (25%) |
| **Self-reported MVPA**^f^ **(PAAQ**^g^**, mins/week)** | 82.9 ± 86.4 | 91.6 ± 84.7 | 71.3 ± 91.1 |

^a^BMI: Body Mass Index.

^b^PHQ-9: Patient Health Questionnaire – 9 item.

^c^MDD: Major depressive disorder.

^d^CBT: Cognitive behavioural therapy.

^e^PA: Physical activity.

^f^MVPA: Moderate-to-vigorous physical activity.

^g^PAAQ: Physical Activity Adults Questionnaire.
