# Supplementary material for: App-Based Physical Activity Intervention for Individuals With Depression (MoodMover): Single-Arm, Pre-Post Proof-of-Concept and Feasibility Study
Source: JMIR Form Res. 2026 Jun 11;10:e79033. doi: 10.2196/79033 (PMC13256492; doi:10.2196/79033)
Supplement: Multimedia Appendix 4 [file formative-v10-e79033-s004.docx]

Multimedia Appendix 4. App engagement, usability, and satisfaction.

|  | Total (N=21) | | | Study completers (n=16) | | | Early dropouts (n=5) | | |
| --- | --- | --- | --- | --- | --- | --- | --- | --- | --- |
| App engagement | Mean ± SD | Min | Max | Mean ± SD | Min | Max | Mean ± SD | Min | Max |
| Total active app use (days) |  |  |  |  |  |  |  |  |  |
| Run-in period (week 1) | 4.0 ± 2.0 | 1 | 7 | 3.9 ± 2.2 | 1 | 7 | 4.2 ± 1.5 | 2 | 6 |
| Intervention period (weeks 2-9) | 30.8 ± 15.2 | 3 | 56 | 37.1 ± 10.8 | 22 | 56 | 10.6 ± 7.6 | 3 | 23 |
| Total active app use duration (mins) | 196.6 ± 108.6 | 24.9 | 391.3 | 229.4 ± 98.0 | 39.7 | 391.3 | 91.5 ± 70.0 | 17.4 | 208.5 |
| Run-in period (week 1) | 27.1 ± 8.7 | 10.2 | 45.7 | 26.8 ± 8.6 | 10.2 | 42.3 | 28.1 ± 9.9 | 22.1 | 45.7 |
| Intervention period (weeks 2-9) | 169.5 ± 106.4 | 1.4 | 357.1 | 202.6 ± 94.2 | 29.5 | 357.1 | 63.4 ± 69.6 | 1.4 | 182.1 |
| Number of major lessons completed | 5.7 ± 2.7 | 0 | 8 | 6.9 ± 1.6 | 4 | 8 | 1.8 ± 1.1 | 0 | 3 |
| Number of complementary lessons completed | 5.5 ± 2.8 | 0 | 8 | 6.8 ± 1.7 | 4 | 8 | 1.6 ± 1.1 | 0 | 3 |
| Time spent on each major lesson (mins) | 8.0 ± 4.7 | 0 | 17.4 | 8.4 ± 4.0 | 2.6 | 15.8 | 6.6 ± 6.6 | 0 | 17.4 |
| Time spent on each complementary lesson (mins) | 3.9 ± 3.0 | 0 | 13.4 | 3.4 ± 2.1 | 0.3 | 7.1 | 5.5 ± 5.1 | 0 | 13.4 |
| Number of times of step syncing | 28.5 ± 17.1 | 0 | 63 | 34.1 ± 15.3 | 0 | 63 | 10.6 ± 7.5 | 0 | 20 |
| Number of surveys completed | 19.7 ± 10.2 | 0 | 28 | 24.6 ± 5.6 | 13 | 28 | 4.2 ± 2.4 | 0 | 6 |
| Number of action plans completed | 6.5 ± 17.8 | 0 | 82 | 8.5 ± 20.2 | 0 | 82 | 0.2 ± 0.4 | 0 | 1 |
| Number of times of step goal adjustments | 2.3 ± 1.5 | 0 | 5 | 2.8 ± 1.3 | 0 | 5 | 0.8 ± 0.8 | 0 | 2 |
| Number of exercise sessions logged | 6.7 ± 9.9 | 0 | 36 | 8.6 ± 10.7 | 0 | 36 | 0.6 ± 0.9 | 0 | 2 |
| Total logged LPA^a^ sessions | 4.5 ± 7.6 | 0 | 29 | 5.8 ± 8.4 | 0 | 29 | 0.4 ± 0.9 | 0 | 2 |
| Total logged MVPA^b^ sessions | 2.2 ± 3.8 | 0 | 16 | 2.9 ± 4.2 | 0 | 16 | 0.2 ± 0.4 | 0 | 1 |
| Total logged LPA minutes | 135.7 ± 176.7 | 0 | 588 | 172.2 ± 187.6 | 0 | 588 | 19.0 ± 42.5 | 0 | 95 |
| Total logged MVPA minutes | 109.1 ± 207.5 | 0 | 884 | 141.9 ± 229.2 | 0 | 884 | 4.0 ± 8.9 | 0 | 20 |
| Mood after LPA sessions | 2.7 ± 0.3 (n=13) | 2.3 | 3.1 | 2.7 ± 0.3 (n=12) | 2.3 | 3.1 | 2.5 (n=1) |  |  |
| Mood after MVPA sessions | 2.8 ± 0.4 (n=11) | 2.0 | 3.5 | 2.9 ± 0.3 (n=10) | 2.5 | 3.5 | 2.0 (n=1) |  |  |
| Overall Mood | 2.7 ± 0.4 (n=16) | 2.0 | 3.5 | 2.8 ± 0.3 (n=14) | 2.4 | 3.5 | 2.3 ± 0.4 (n=2) | 2.0 | 2.5 |
| MAUQ^c^ |  |  |  | 5.01 ± 1.02 | 3.23 | 6.92 |  |  |  |
| Satisfaction |  |  |  | 53.56 ± 8.11 | 36 | 66 |  |  |  |

^a^LPA: Light physical activity.

^b^MVPA: Moderate-to-vigorous physical activity.

^c^MAUQ: mHealth App Usability Questionnaire.
