# Supplementary material for: App-Based Physical Activity Intervention for Individuals With Depression (MoodMover): Single-Arm, Pre-Post Proof-of-Concept and Feasibility Study
Source: JMIR Form Res. 2026 Jun 11;10:e79033. doi: 10.2196/79033 (PMC13256492; doi:10.2196/79033)
Supplement: Multimedia Appendix 9 [file formative-v10-e79033-s009.docx]

Multimedia Appendix 9. Correlations between changes in PA^a^ and PHQ-9^b^

|  | **Correlations** | **75% CI** | **85% CI** | **95% CI** |
| --- | --- | --- | --- | --- |
| **Smartphone-based step counts** |  |  |  |  |
| Pearson's | r = -0.55 | **[-0.75, -0.27]** | **[-0.78, -0.18]** | **[-0.84, -0.03]** |
| Spearman's | ρ = -0.29 | [-0.59, 0.06] | [-0.66, 0.15] | [-0.76, 0.31] |
| **Self-reported MVPA^c^** |  |  |  |  |
| Pearson's | r = -0.77 | **[-0.87, -0.60]** | **[-0.89, -0.54]** | **[-0.91, -0.44]** |
| Spearman's | ρ = -0.85 | **[-0.91, -0.73]** | **[-0.93, -0.69]** | **[-0.95, -0.61]** |
| **Self-reported MVPA (no outlier)** |  |  |  |  |
| Pearson's | r = -0.80 | **[-0.89, -0.65]** | **[-0.91, -0.60]** | **[-0.93, -0.49]** |
| Spearman's | ρ = -0.82 | **[-0.89, -0.69]** | **[-0.91, -0.65]** | **[-0.94, -0.55]** |

^a^PA: Physical activity.

^b^PHQ-9: Patient Health Questionnaire – 9 item.

^c^MVPA: Moderate-to-vigorous physical activity.
