# Supplementary material for: App-Based Physical Activity Intervention for Individuals With Depression (MoodMover): Single-Arm, Pre-Post Proof-of-Concept and Feasibility Study
Source: JMIR Form Res. 2026 Jun 11;10:e79033. doi: 10.2196/79033 (PMC13256492; doi:10.2196/79033)
Supplement: Multimedia Appendix 10 [file formative-v10-e79033-s010.docx]

Multimedia Appendix 10. ChatGPT transcripts

Authors: Please create a line chart showing the following data of weekly app use duration (in minutes) and their standard deviations over the 8-week intervention phase. Please label the x-axis as "Week" and the y-axis as "Mean app use duration (mins)."

ChatGPT: Figure 2.

Authors: Please create a heatmap of the following data of weekly step count data over 9 weeks. Label the x-axis as "Week" and the y-axis as "Participant." Include a scale on the right side of the figure to illustrate the range of step counts. Highlight the participant who increased their daily steps by 3,000 at week 9 compared to baseline with a red rectangle.

ChatGPT: Figure 3.
