# Supplementary material for: App-Based Physical Activity Intervention for Individuals With Depression (MoodMover): Single-Arm, Pre-Post Proof-of-Concept and Feasibility Study
Source: JMIR Form Res. 2026 Jun 11;10:e79033. doi: 10.2196/79033 (PMC13256492; doi:10.2196/79033)
Supplement: Checklist 1 [file formative-v10-e79033-s011.docx]

Multimedia Appendix 1. STROBE Statement—Checklist of items that should be included in reports of ***cohort studies***

|  | Item no. | Recommendation | Page no. |
| --- | --- | --- | --- |
| Title and abstract | 1 | (a) Indicate the study’s design with a commonly used term in the title or the abstract |  |
|  |  | (b) Provide in the abstract an informative and balanced summary of what was done and what was found | 1-2 |
| Introduction | | | |
| Background/rationale | 2 | Explain the scientific background and rationale for the investigation being reported | 3-4 |
| Objectives | 3 | State specific objectives, including any prespecified hypotheses | 4-5 |
| Methods | | | |
| Study design | 4 | Present key elements of study design early in the paper | 5 |
| Setting | 5 | Describe the setting, locations, and relevant dates, including periods of recruitment, exposure, follow-up, and data collection | 6-7 |
| Participants | 6 | (a) Give the eligibility criteria, and the sources and methods of selection of participants. Describe methods of follow-up | 6 |
|  |  | (b) For matched studies, give matching criteria and number of exposed and unexposed |  |
| Variables | 7 | Clearly define all outcomes, exposures, predictors, potential confounders, and effect modifiers. Give diagnostic criteria, if applicable | 8-12 |
| Data sources/ measurement | 8* | For each variable of interest, give sources of data and details of methods of assessment (measurement). Describe comparability of assessment methods if there is more than one group | 8-12 |
| Bias | 9 | Describe any efforts to address potential sources of bias | 7 |
| Study size | 10 | Explain how the study size was arrived at | 12 |
| Quantitative variables | 11 | Explain how quantitative variables were handled in the analyses. If applicable, describe which groupings were chosen and why | 12-13 |
| Statistical methods | 12 | (a) Describe all statistical methods, including those used to control for confounding | 12-13 |
|  |  | (b) Describe any methods used to examine subgroups and interactions |  |
|  |  | (c) Explain how missing data were addressed |  |
|  |  | (d) If applicable, explain how loss to follow-up was addressed |  |
|  |  | (e) Describe any sensitivity analyses |  |
| Results | | |  |
| Participants | 13* | (a) Report numbers of individuals at each stage of study—eg numbers potentially eligible, examined for eligibility, confirmed eligible, included in the study, completing follow-up, and analysed | 13-14; Figure 1 |
|  |  | (b) Give reasons for non-participation at each stage |  |
|  |  | (c) Consider use of a flow diagram |  |
| Descriptive data | 14* | (a) Give characteristics of study participants (eg demographic, clinical, social) and information on exposures and potential confounders | 15; Multimedia Appendix 4 |
|  |  | (b) Indicate number of participants with missing data for each variable of interest |  |
|  |  | (c) Summarise follow-up time (eg, average and total amount) |  |
| Outcome data | 15* | Report numbers of outcome events or summary measures over time | 15-21 |
| Other analyses | 17 | Report other analyses done—eg analyses of subgroups and interactions, and sensitivity analyses | 21; Multimedia Appendix 5,8,10 |
| Discussion | | | |
| Key results | 18 | Summarise key results with reference to study objectives | 21-27 |
| Limitations | 19 | Discuss limitations of the study, taking into account sources of potential bias or imprecision. Discuss both direction and magnitude of any potential bias | 27-29 |
| Interpretation | 20 | Give a cautious overall interpretation of results considering objectives, limitations, multiplicity of analyses, results from similar studies, and other relevant evidence | 29 |
| Generalisability | 21 | Discuss the generalisability (external validity) of the study results | NA |
| Other information | | | |
| Funding | 22 | Give the source of funding and the role of the funders for the present study and, if applicable, for the original study on which the present article is based | 29 |

*Give information separately for exposed and unexposed groups.

**Note:** An Explanation and Elaboration article discusses each checklist item and gives methodological background and published examples of transparent reporting. The STROBE checklist is best used in conjunction with this article (freely available on the Web sites of PLoS Medicine at http://www.plosmedicine.org/, Annals of Internal Medicine at http://www.annals.org/, and Epidemiology at http://www.epidem.com/). Information on the STROBE Initiative is available at http://www.strobe-statement.org.

**2. CONSORT 2020 statement extension to pilot and feasibility trials (modified for single-arm trial).**

| **Section/topic and item no.** | **Extension for single-arm pilot trials item descriptor** | **Page no.** |
| --- | --- | --- |
| **Introduction** |  |  |
| *Background and objectives* |  |  |
| 2a | Scientific background and explanation of rationale for future definitive trial, and reasons for a single-arm pilot trial | 3-4 |
| 2b | Specific objectives or research questions for single-arm pilot trial | 4-5 |
| **Methods** |  |  |
| *Outcomes* |  |  |
| 6a | Completely defined prespecified assessments or measurements to address each pilot trial objective specified in 2b, including how and when they were assessed | 8-12 |
| *Sample size* |  |  |
| 7a | Rationale for numbers in the single-arm pilot trial | 12 |
| *Analytical methods* |  |  |
| 12a | Methods used to address each pilot trial objective whether qualitative or quantitative | 12-13 |
| **Results** |  |  |
| *Numbers analysed*  *16* | For each objective, number of participants (denominator) included in each analysis. If relevant, these numbers  should be by randomised group | 15-19 |
| *Outcomes and estimation*  *17* | For each objective, results including expressions of uncertainty (such as 95% confidence interval) for any  estimates. If relevant, these results should be by randomised group | 19-22; Table 1, Multimedia Appendix 5, 6, 8, 9 |
| **Discussion** |  |  |
| *Interpretation*  *22* | Interpretation consistent with feasibility trial objectives and findings, balancing potential benefits and harms, and  considering other relevant evidence | 22-30 |
| *22a* | Implications for progression from feasibility to future definitive trial, including any proposed amendments | 22-30 |
| **Other information** |  |  |
| *Registration*  *23* | Registration number for feasibility trial and name of trial registry | 2 |
| *Protocol*  *24* | Where the feasibility trial protocol can be accessed, if available | 5 |
